# Supplementary figures and images for: DLG1 functions upstream of SDCCAG3 and IFT20 to control ciliary targeting of polycystin-2
Source: EMBO Rep. 2024 Jun 7;25(7):3040–63. doi: 10.1038/s44319-024-00170-1 (PMC11239879; doi:10.1038/s44319-024-00170-1)

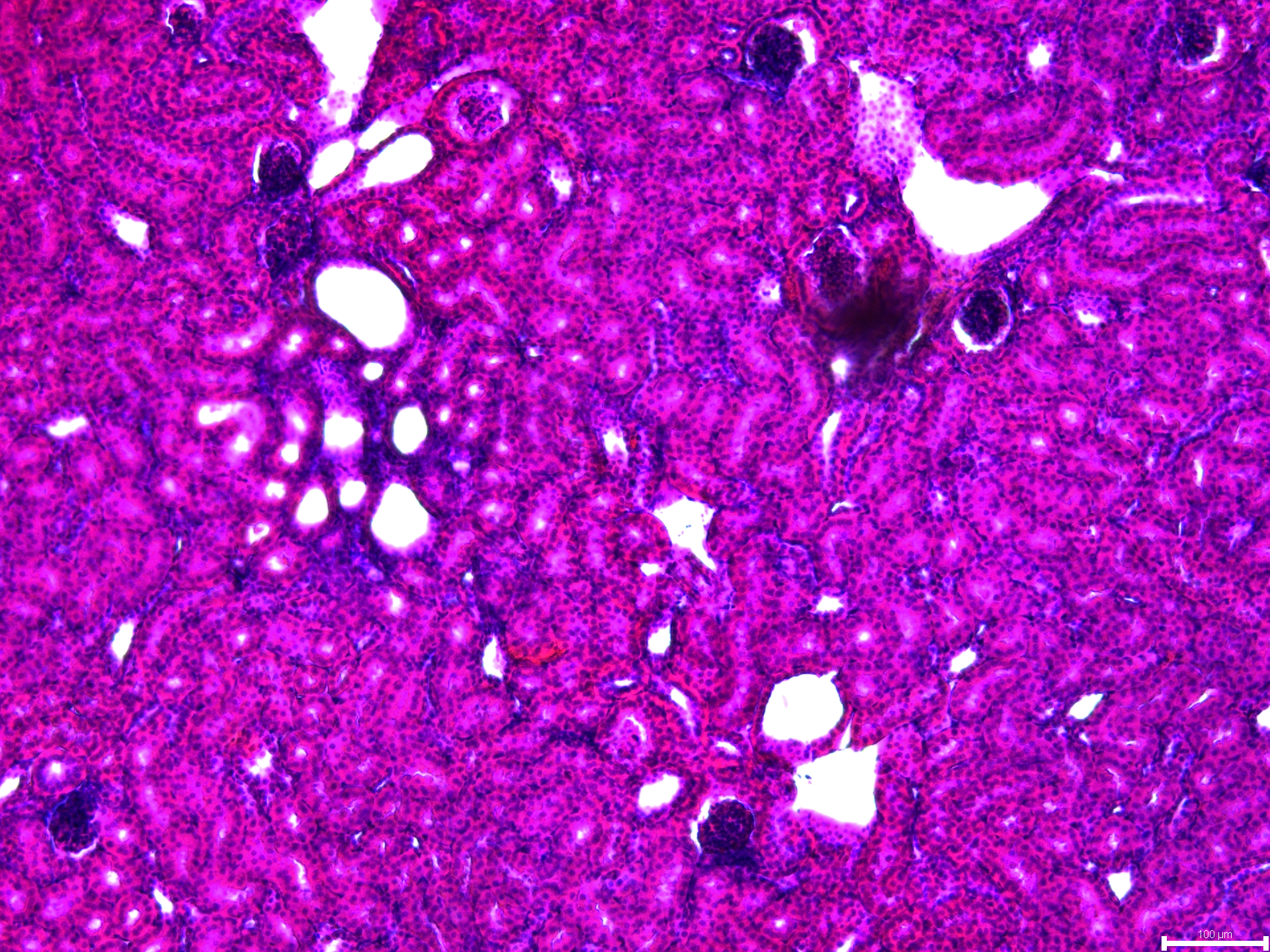

Supplement: Supplementary file 4 — Source data Fig. 1 [file 44319_2024_170_MOESM4_ESM.zip › Figure 1 Source Data/Panel 1A/Pax3Cre-Dlg1FF-inset-10x.tif]

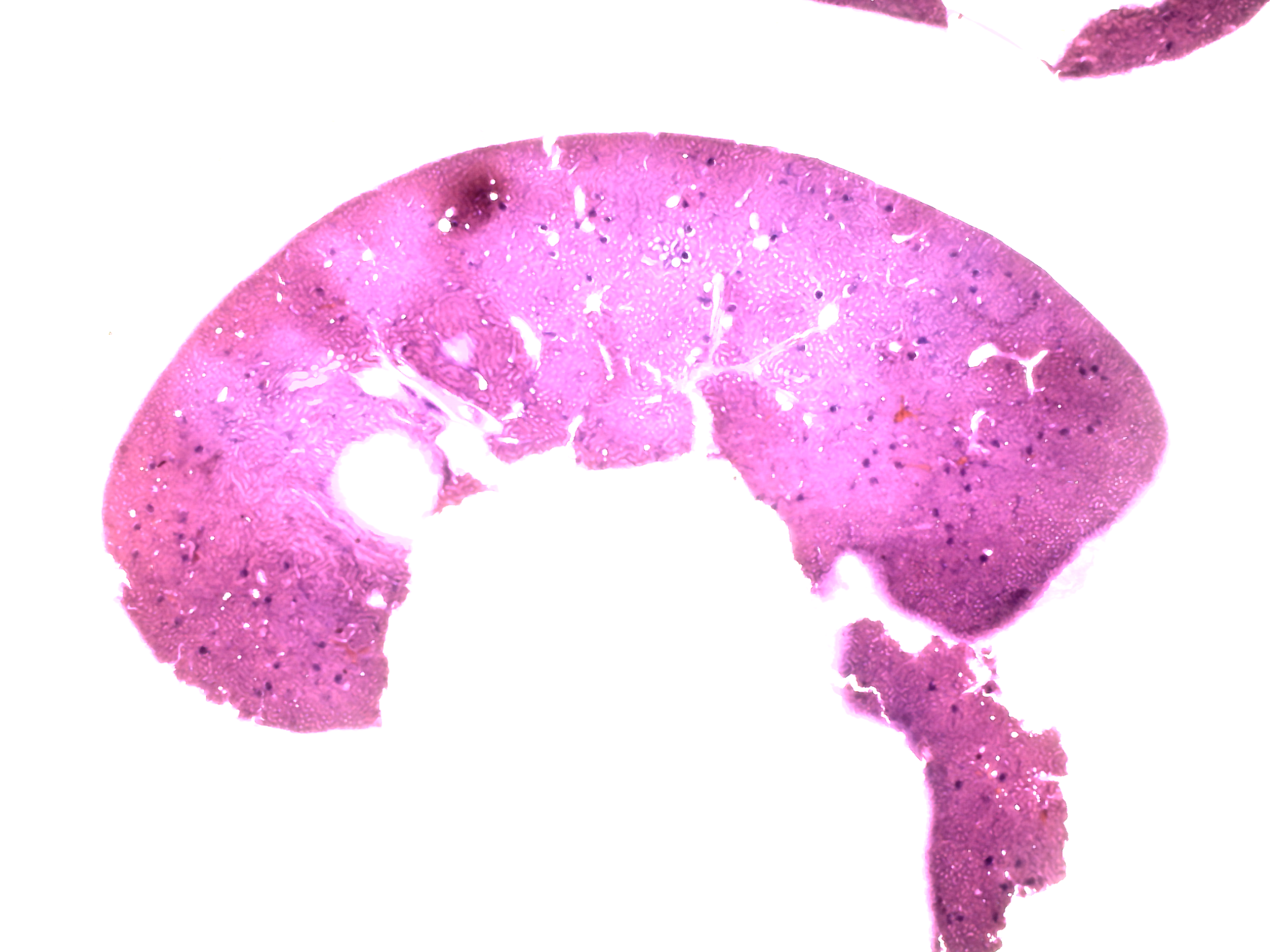

Supplement: Supplementary file 4 — Source data Fig. 1 [file 44319_2024_170_MOESM4_ESM.zip › Figure 1 Source Data/Panel 1A/Pax3Cre-Dlg1FF-left-2x.tif]

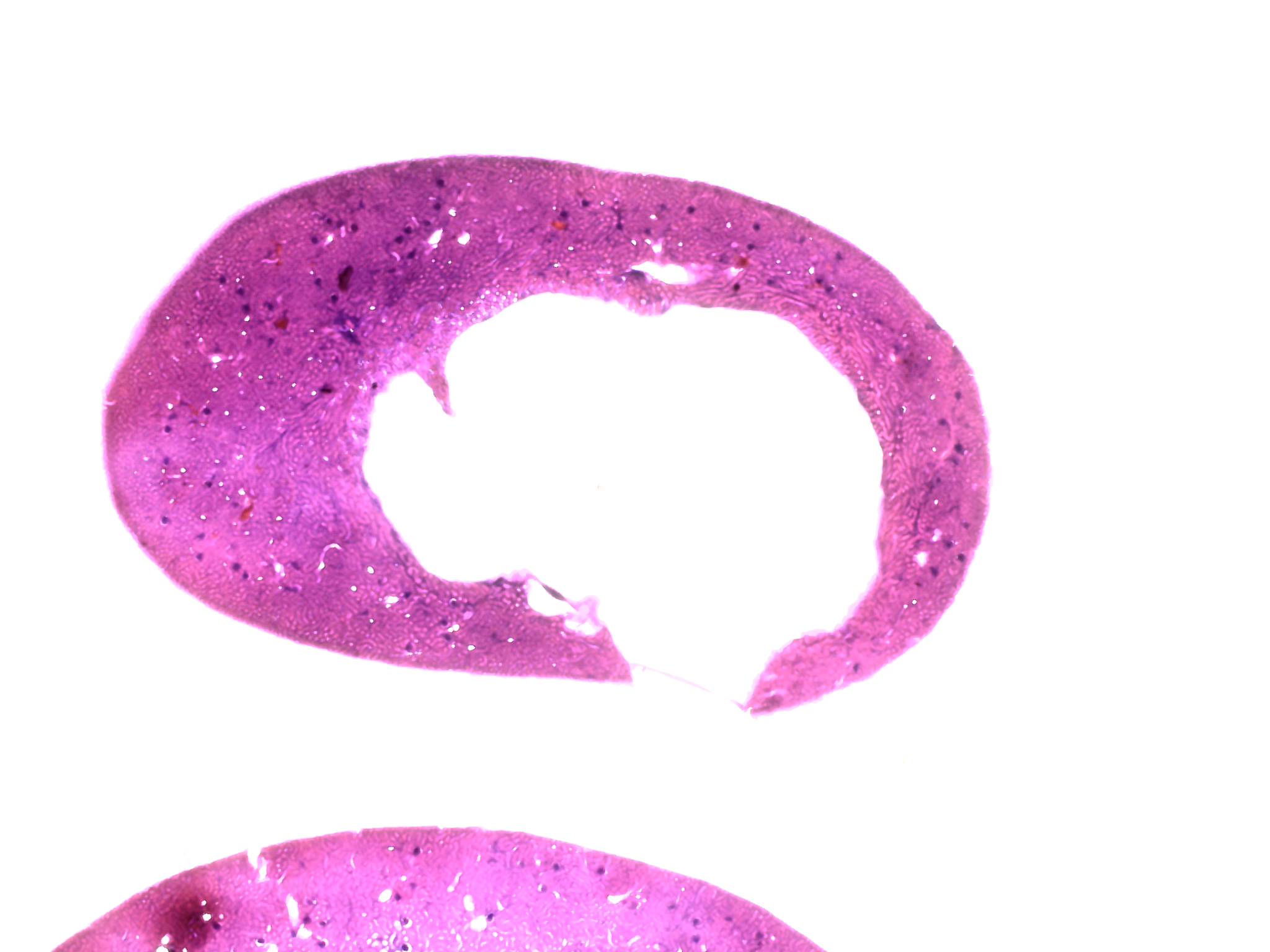

Supplement: Supplementary file 4 — Source data Fig. 1 [file 44319_2024_170_MOESM4_ESM.zip › Figure 1 Source Data/Panel 1A/Pax3Cre-Dlg1FF-right-2x.tif]

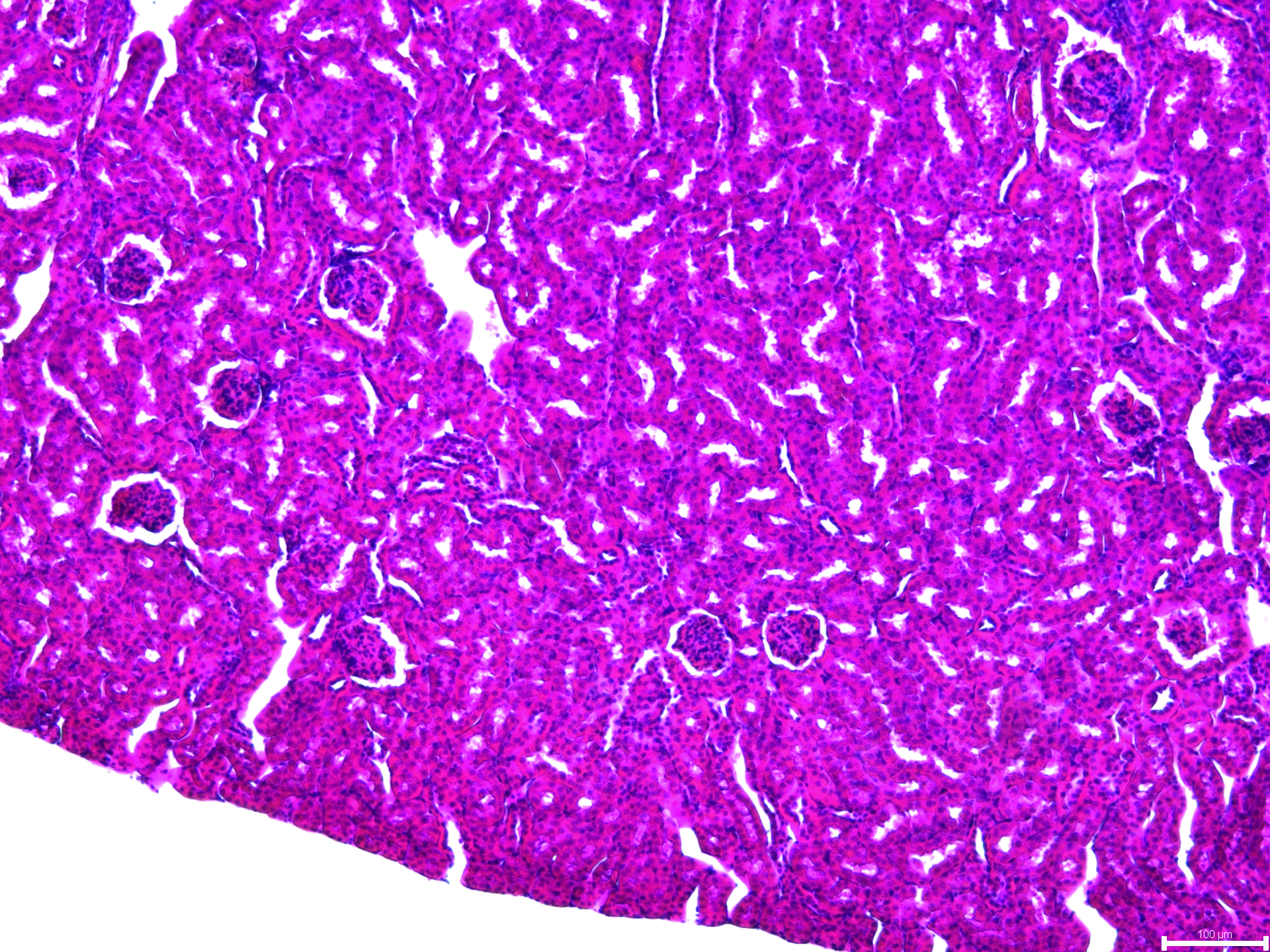

Supplement: Supplementary file 4 — Source data Fig. 1 [file 44319_2024_170_MOESM4_ESM.zip › Figure 1 Source Data/Panel 1A/Wildtype-inset-10x.tif]

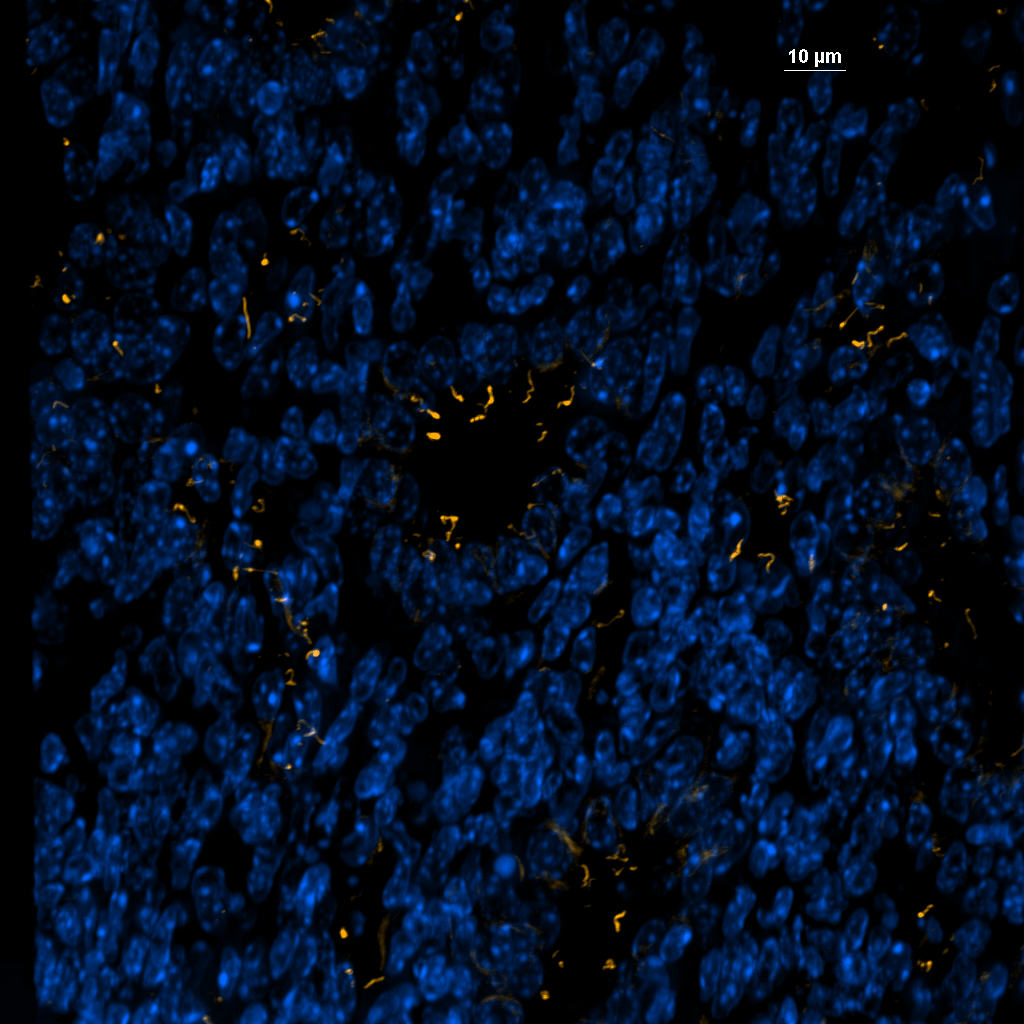

Supplement: Supplementary file 4 — Source data Fig. 1 [file 44319_2024_170_MOESM4_ESM.zip › Figure 1 Source Data/Panel 1B/Pax3Cre-Dlg1FF-Focused Images_RGB.tif]

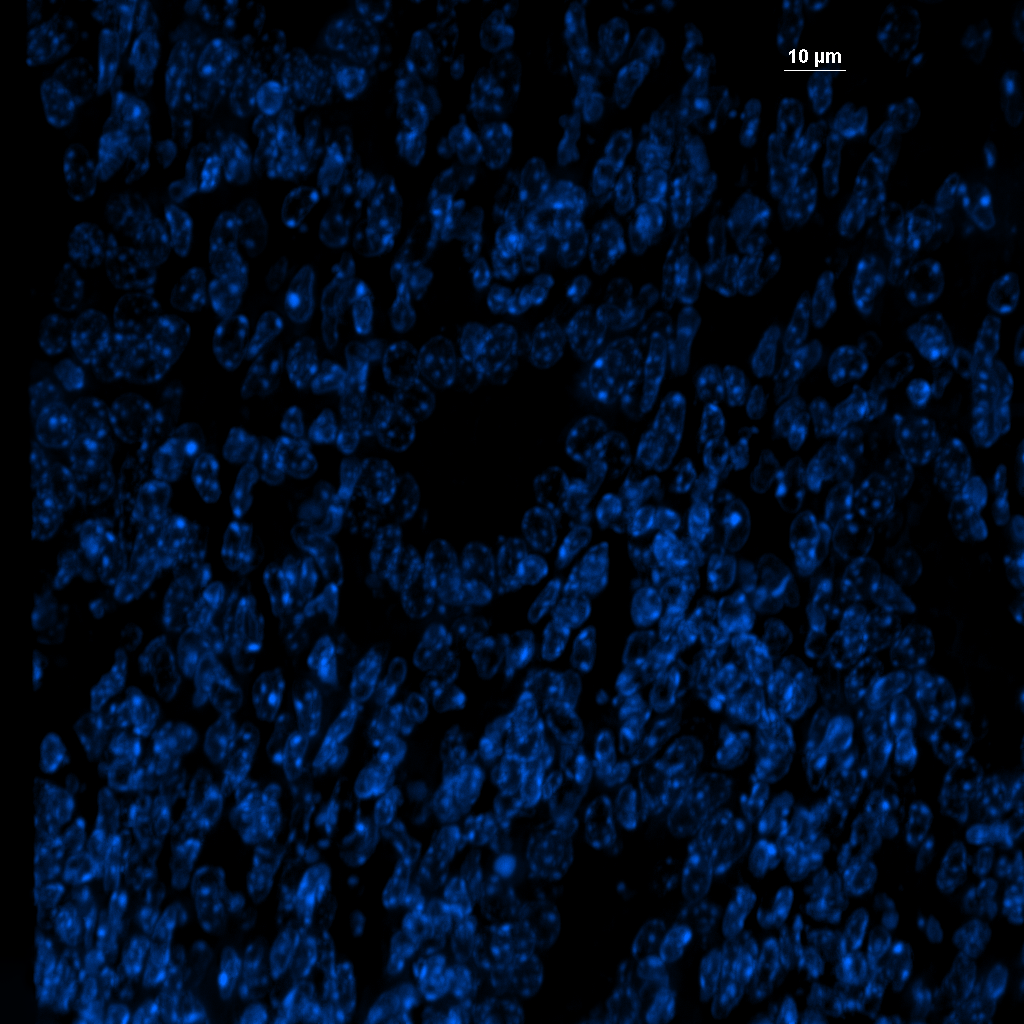

Supplement: Supplementary file 4 — Source data Fig. 1 [file 44319_2024_170_MOESM4_ESM.zip › Figure 1 Source Data/Panel 1B/Pax3Cre-Dlg1FF-Focused Images-RGB_405-SD .tif]

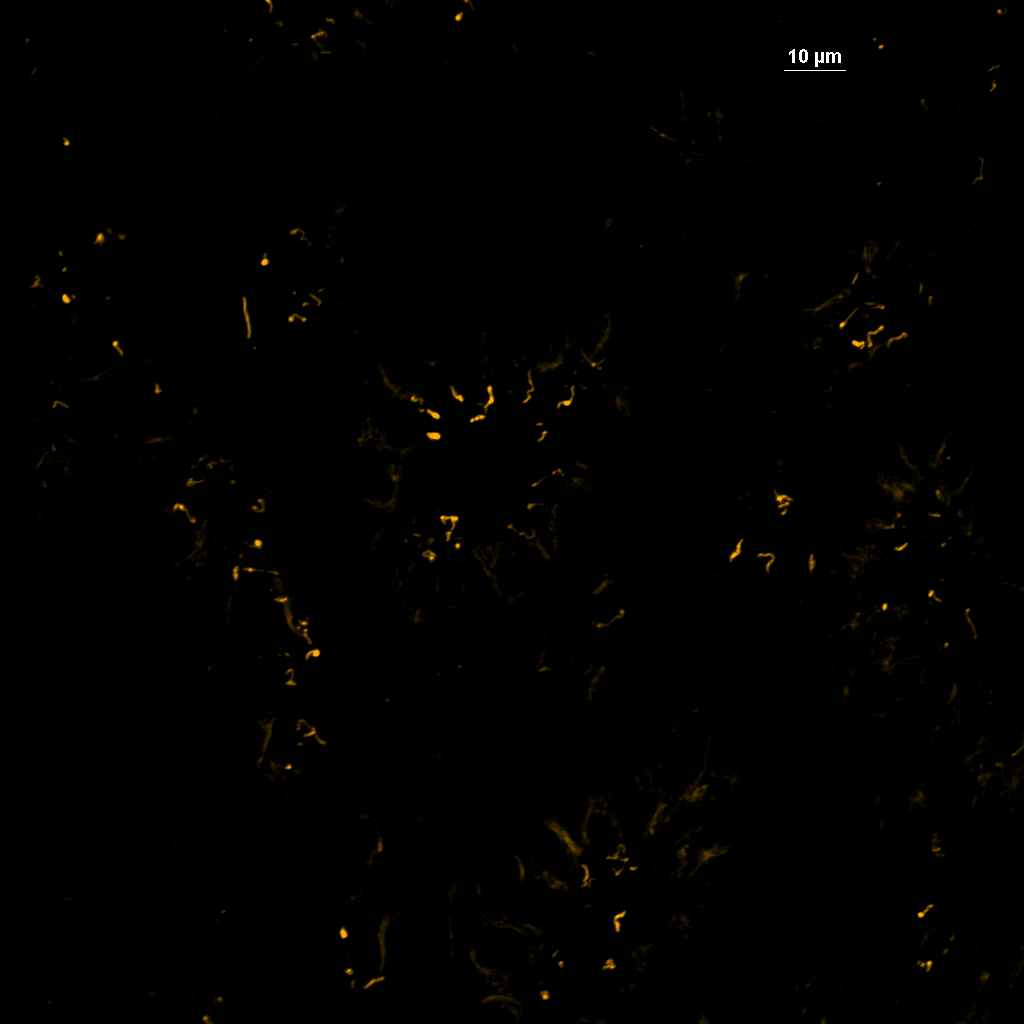

Supplement: Supplementary file 4 — Source data Fig. 1 [file 44319_2024_170_MOESM4_ESM.zip › Figure 1 Source Data/Panel 1B/Pax3Cre-Dlg1FF-Focused Images-RGB_561-SD.tif]

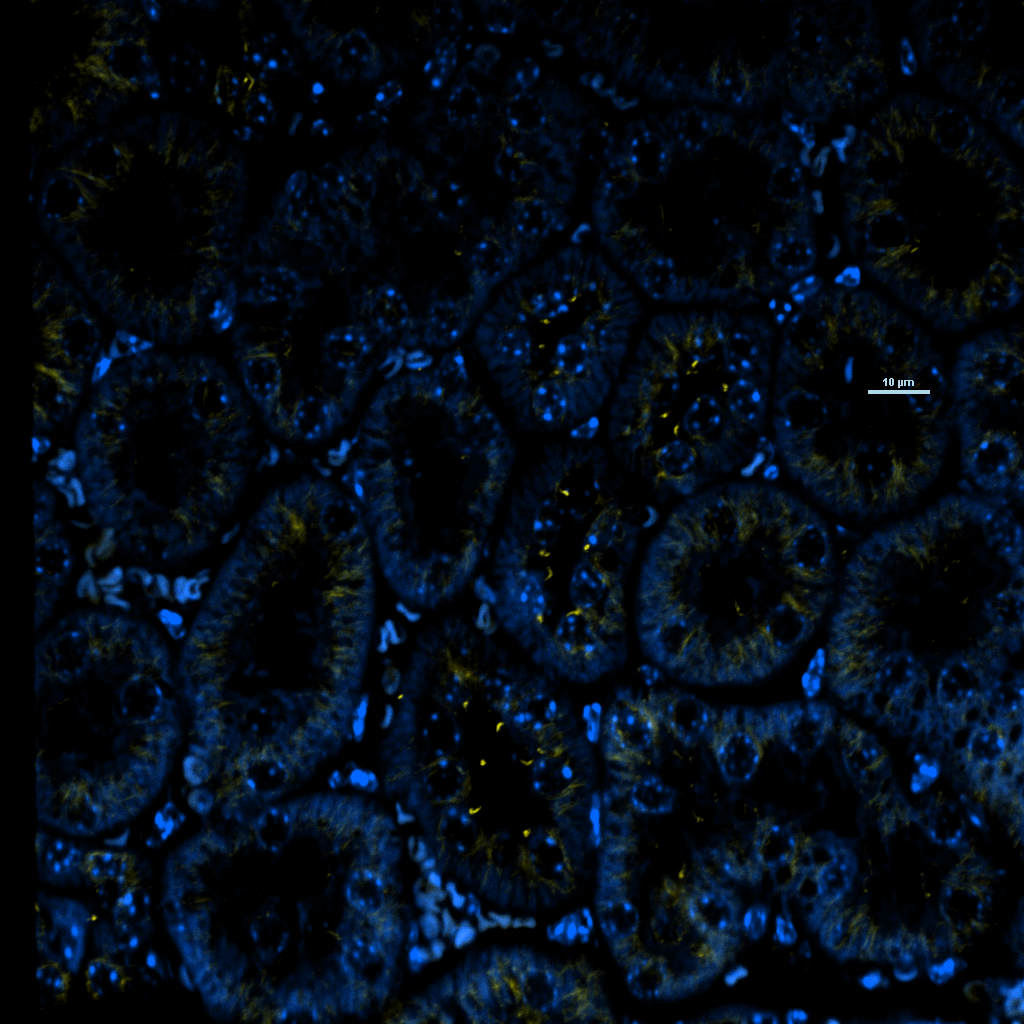

Supplement: Supplementary file 4 — Source data Fig. 1 [file 44319_2024_170_MOESM4_ESM.zip › Figure 1 Source Data/Panel 1B/Wildtype-Focused Images_RGB.tif]

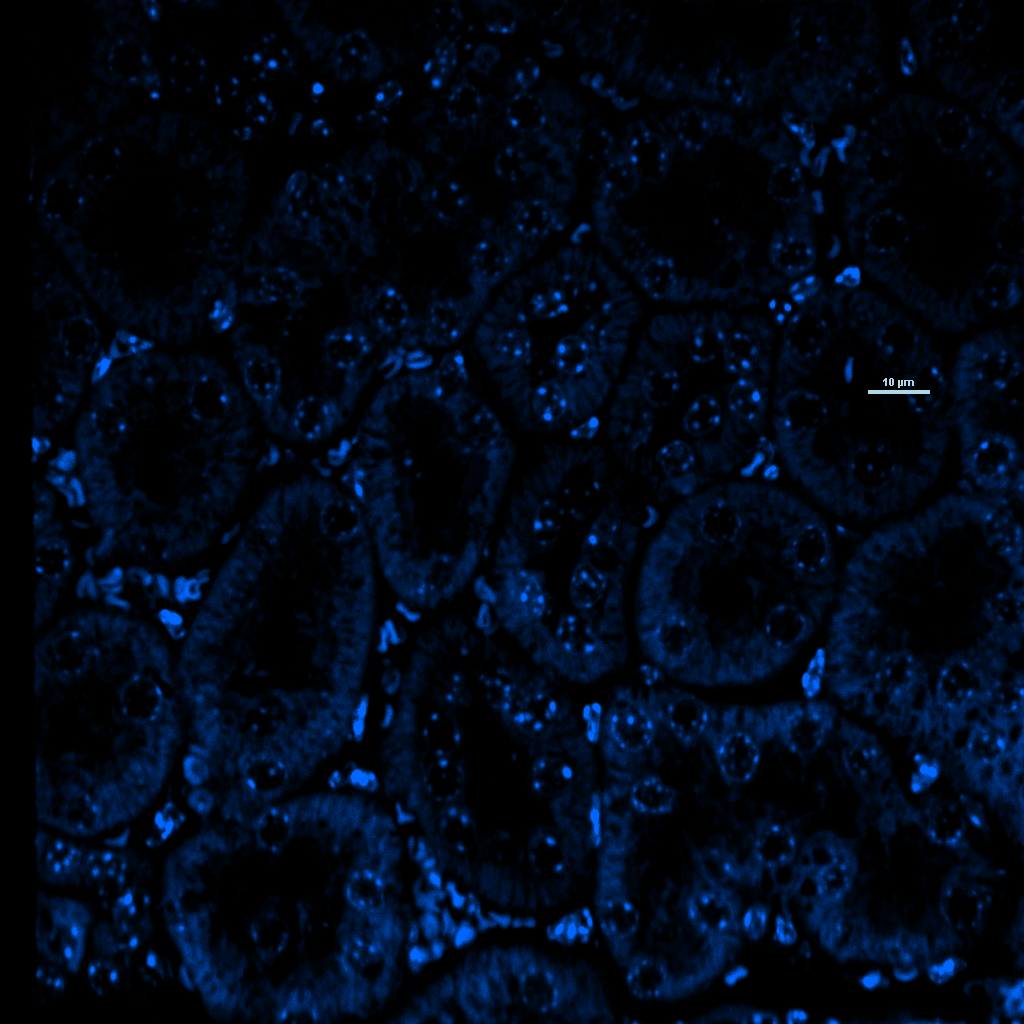

Supplement: Supplementary file 4 — Source data Fig. 1 [file 44319_2024_170_MOESM4_ESM.zip › Figure 1 Source Data/Panel 1B/Wildtype-Focused Images_RGB_405-SD .tif]

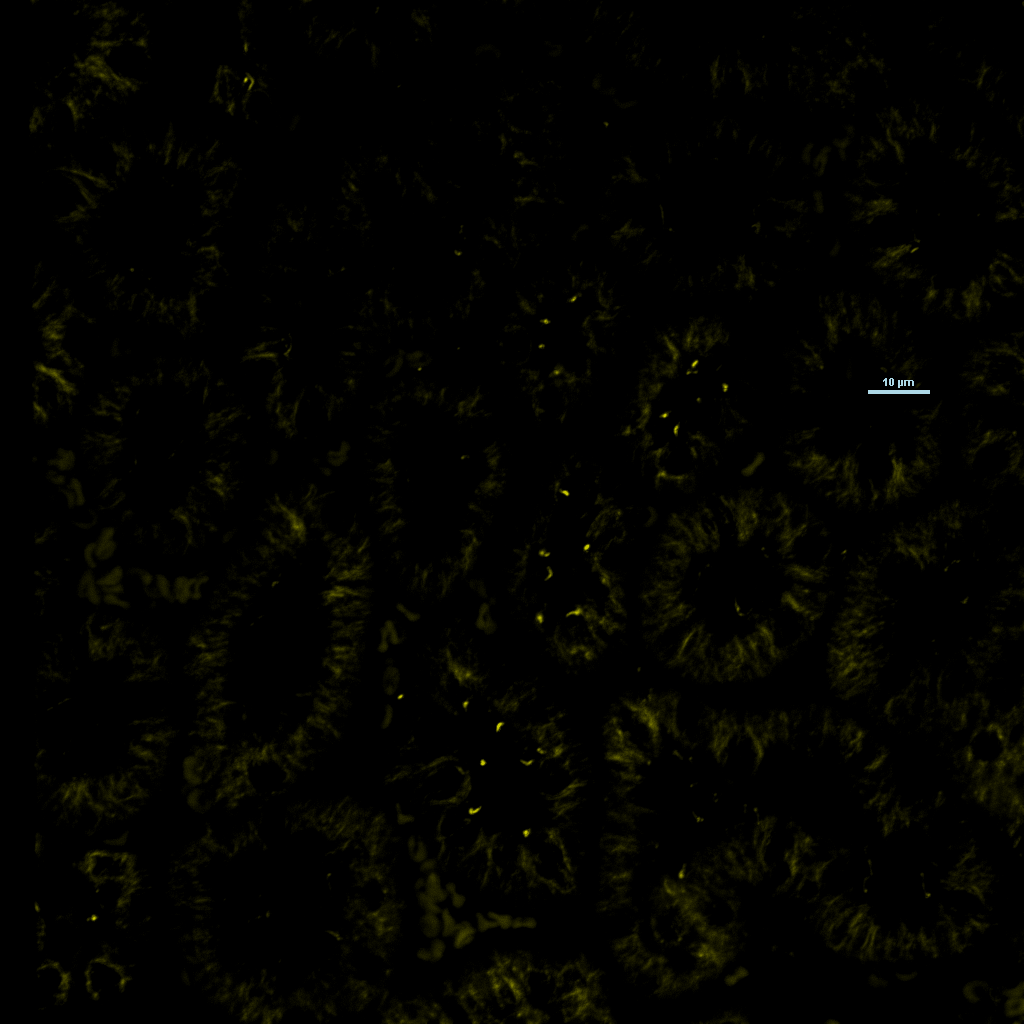

Supplement: Supplementary file 4 — Source data Fig. 1 [file 44319_2024_170_MOESM4_ESM.zip › Figure 1 Source Data/Panel 1B/Wildtype-Focused Images_RGB_561-SD.tif]

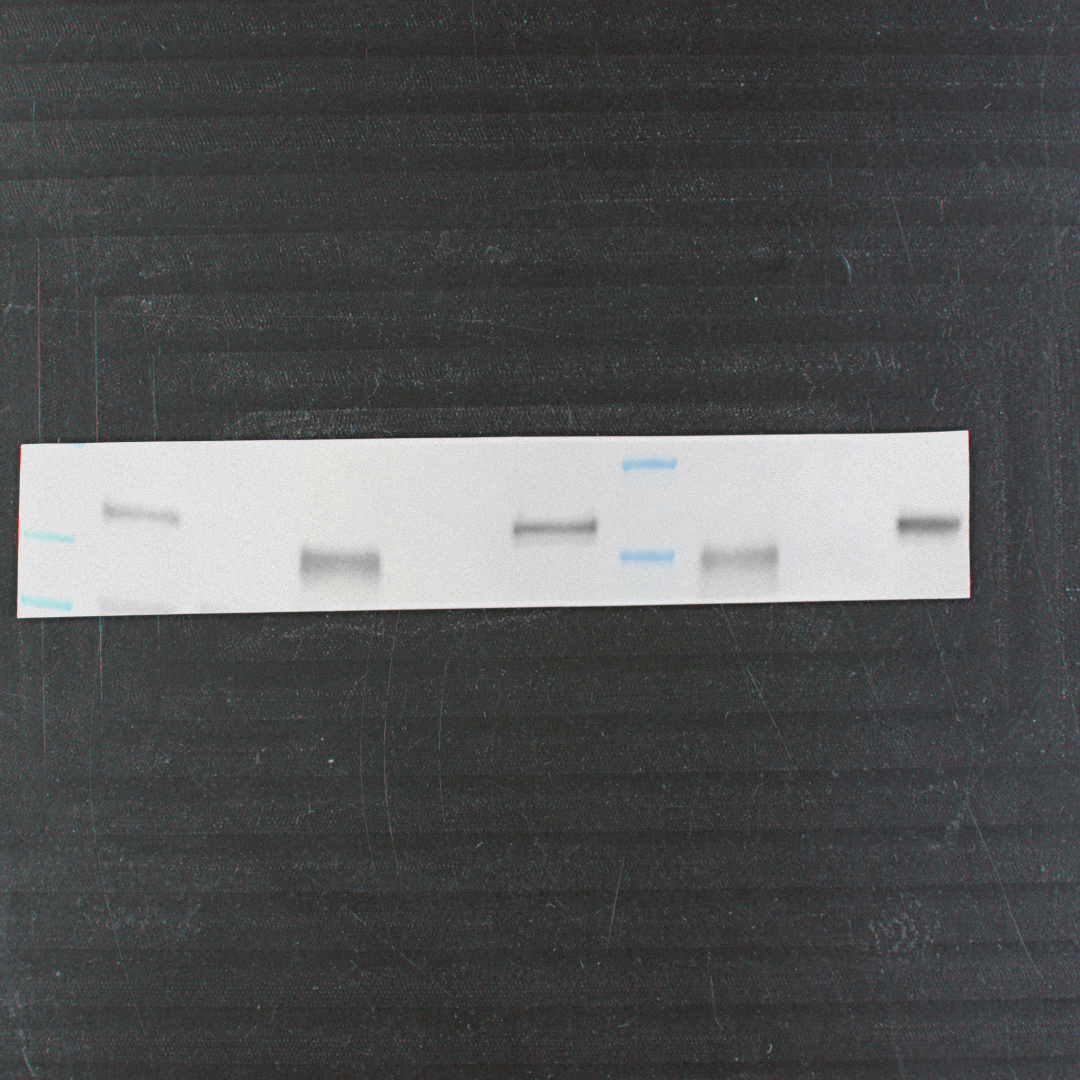

Supplement: Supplementary file 4 — Source data Fig. 1 [file 44319_2024_170_MOESM4_ESM.zip › Figure 1 Source Data/Panel 1F/DLG1_with-ladder.Tif]

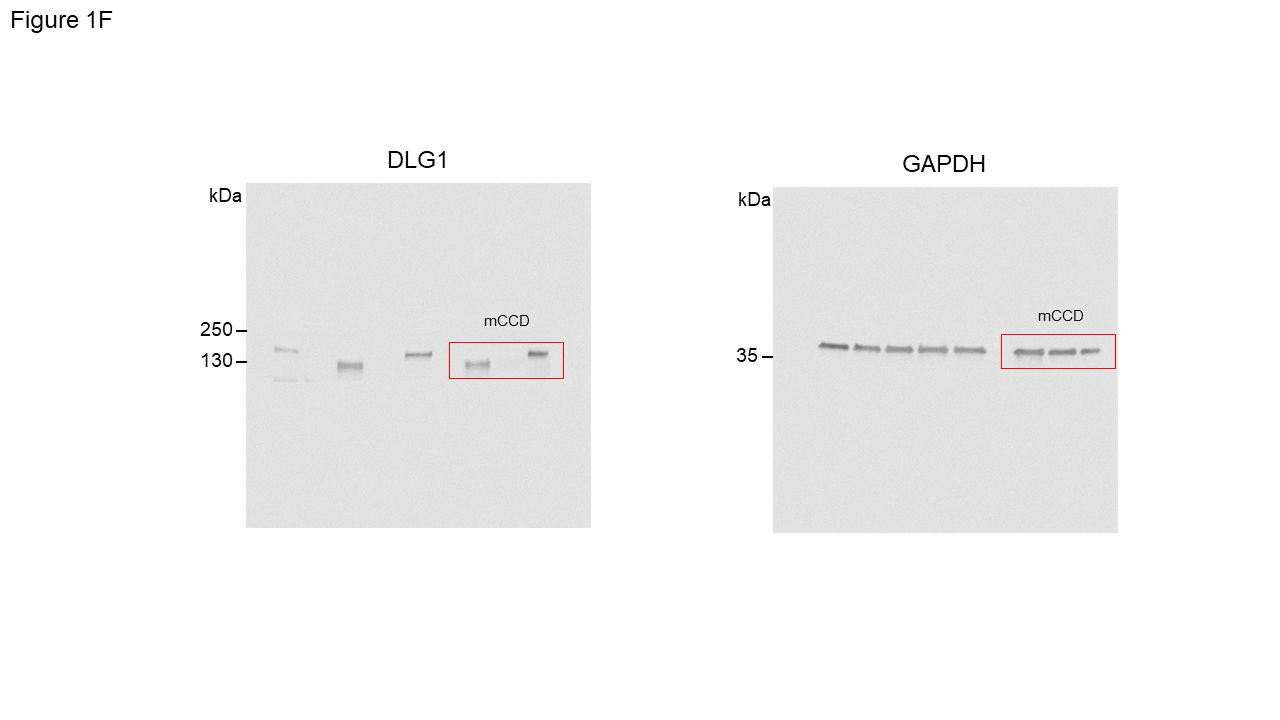

Supplement: Supplementary file 4 — Source data Fig. 1 [file 44319_2024_170_MOESM4_ESM.zip › Figure 1 Source Data/Panel 1F/figure1F_labeling.tif]

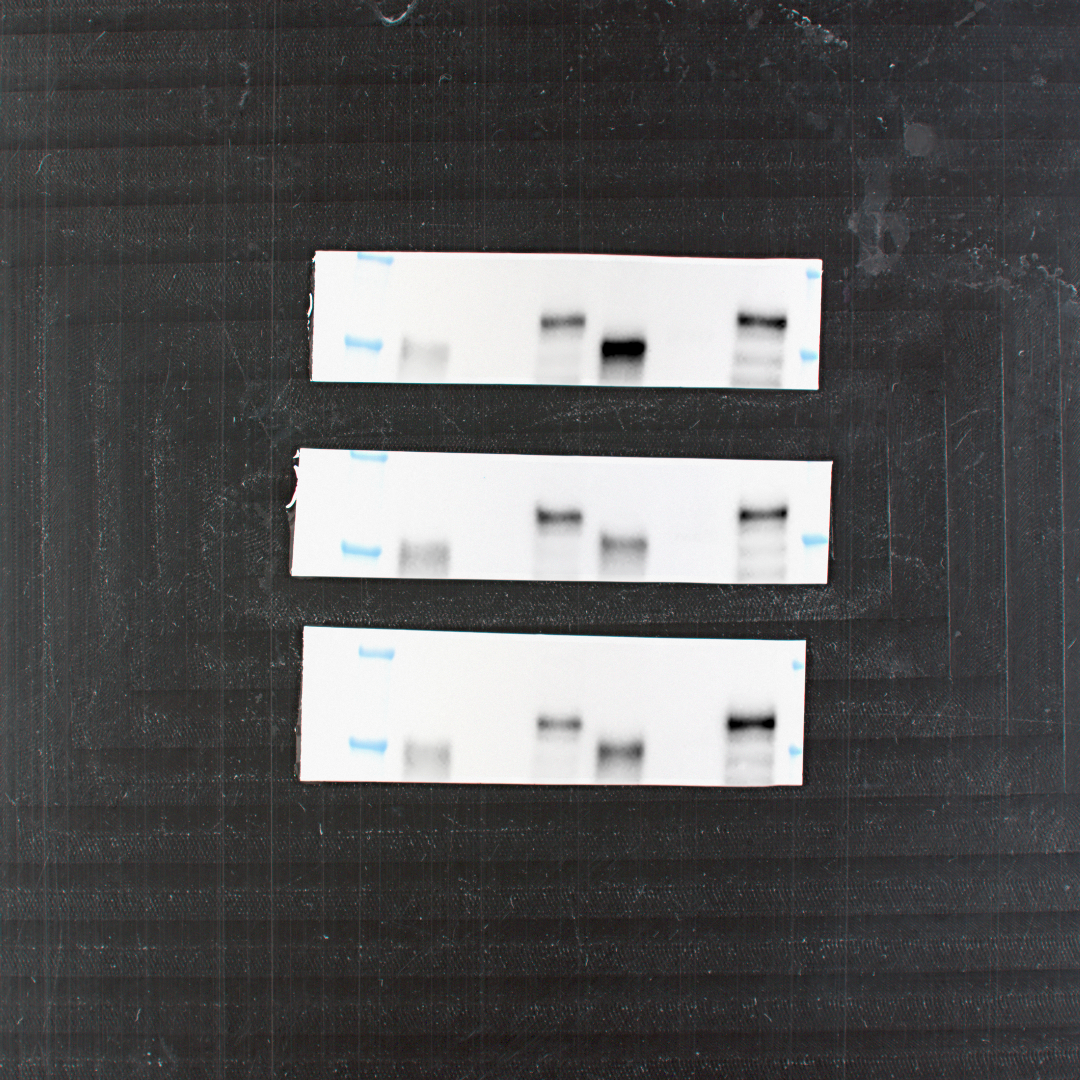

Supplement: Supplementary file 5 — Source data Fig. 3 [file 44319_2024_170_MOESM5_ESM.zip › Figure 3 Source Data/Panel 3C/DLG1_with-ladder.Tif]

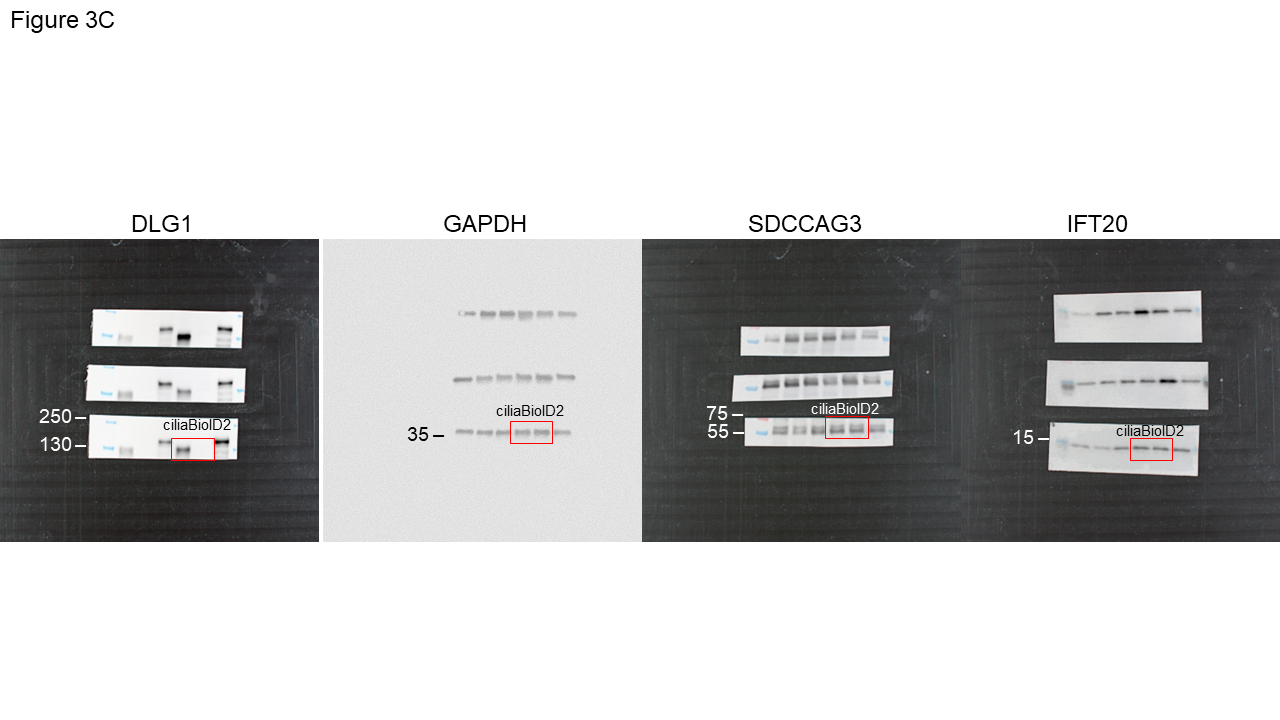

Supplement: Supplementary file 5 — Source data Fig. 3 [file 44319_2024_170_MOESM5_ESM.zip › Figure 3 Source Data/Panel 3C/figure3C_labeling.tif]

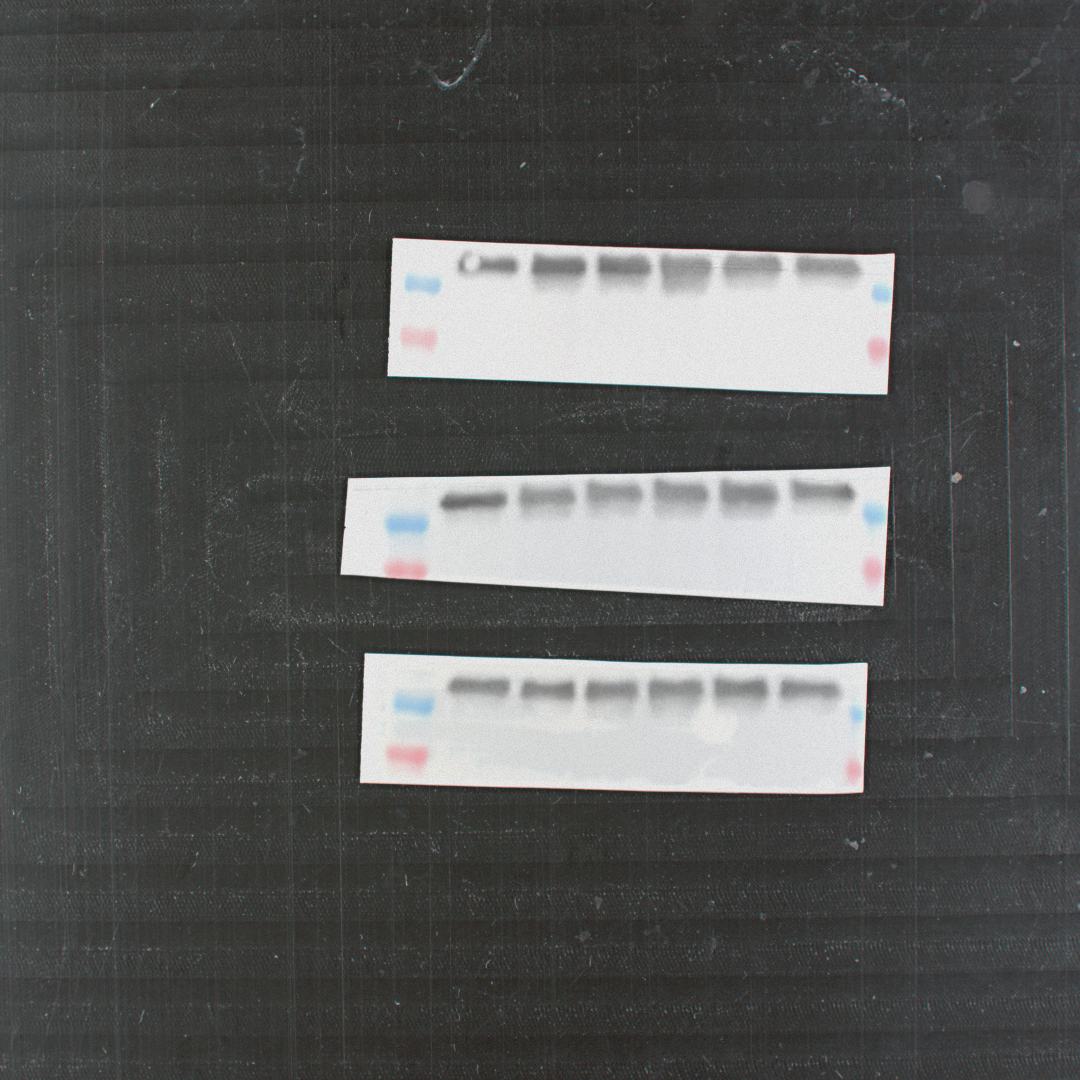

Supplement: Supplementary file 5 — Source data Fig. 3 [file 44319_2024_170_MOESM5_ESM.zip › Figure 3 Source Data/Panel 3C/GAPDH_with-ladder.Tif]

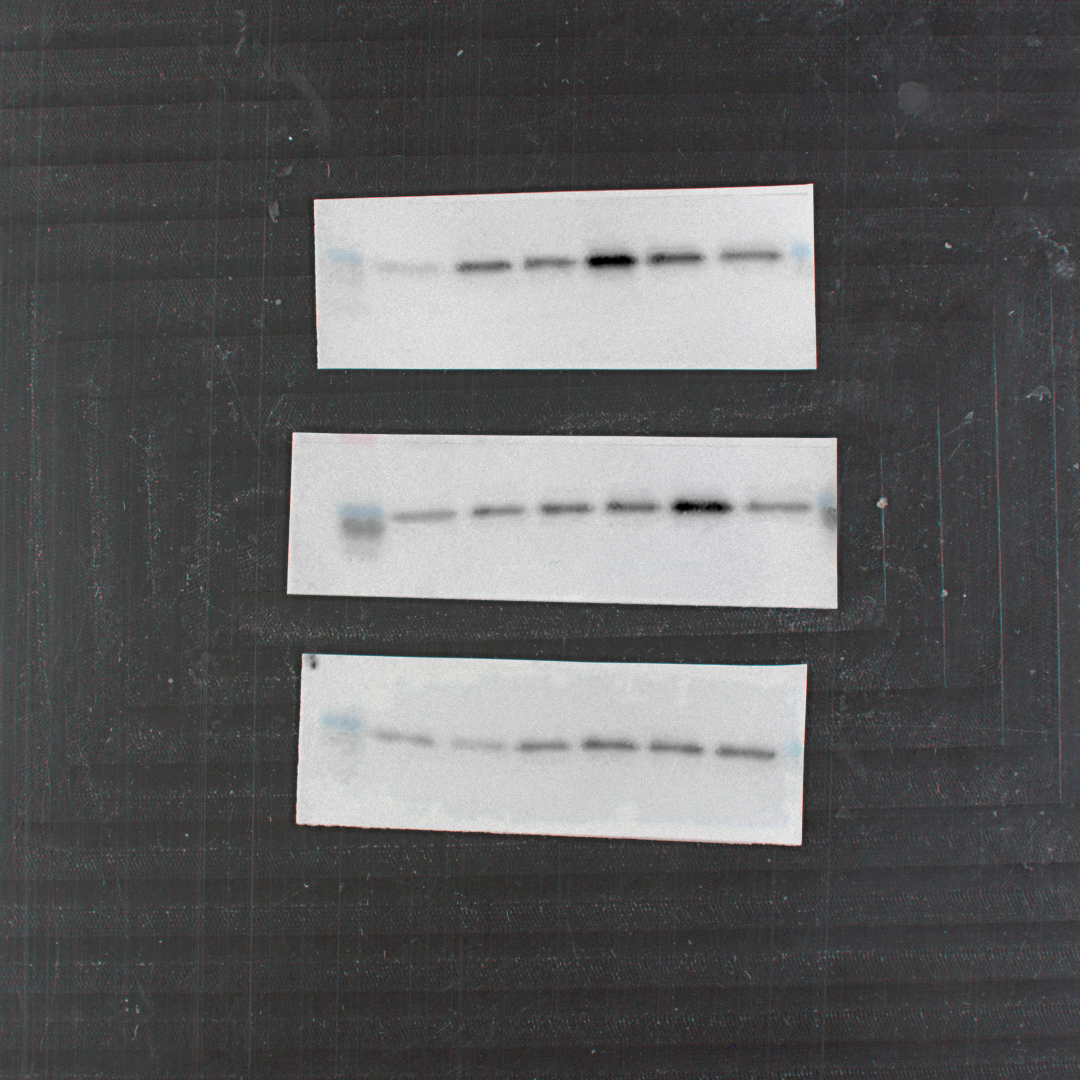

Supplement: Supplementary file 5 — Source data Fig. 3 [file 44319_2024_170_MOESM5_ESM.zip › Figure 3 Source Data/Panel 3C/IFT20_with-ladder.Tif]

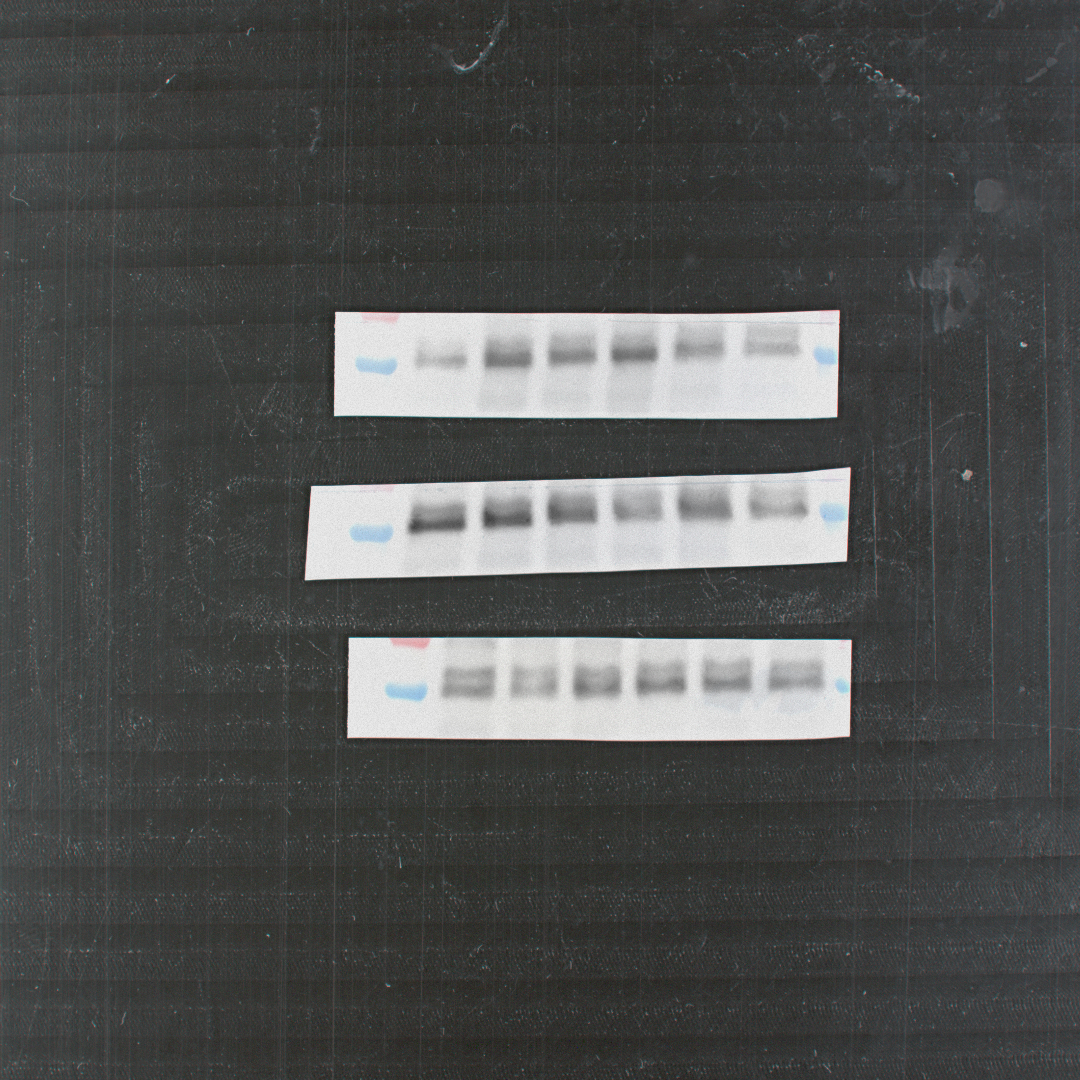

Supplement: Supplementary file 5 — Source data Fig. 3 [file 44319_2024_170_MOESM5_ESM.zip › Figure 3 Source Data/Panel 3C/SDCCAG3_with-ladder.Tif]

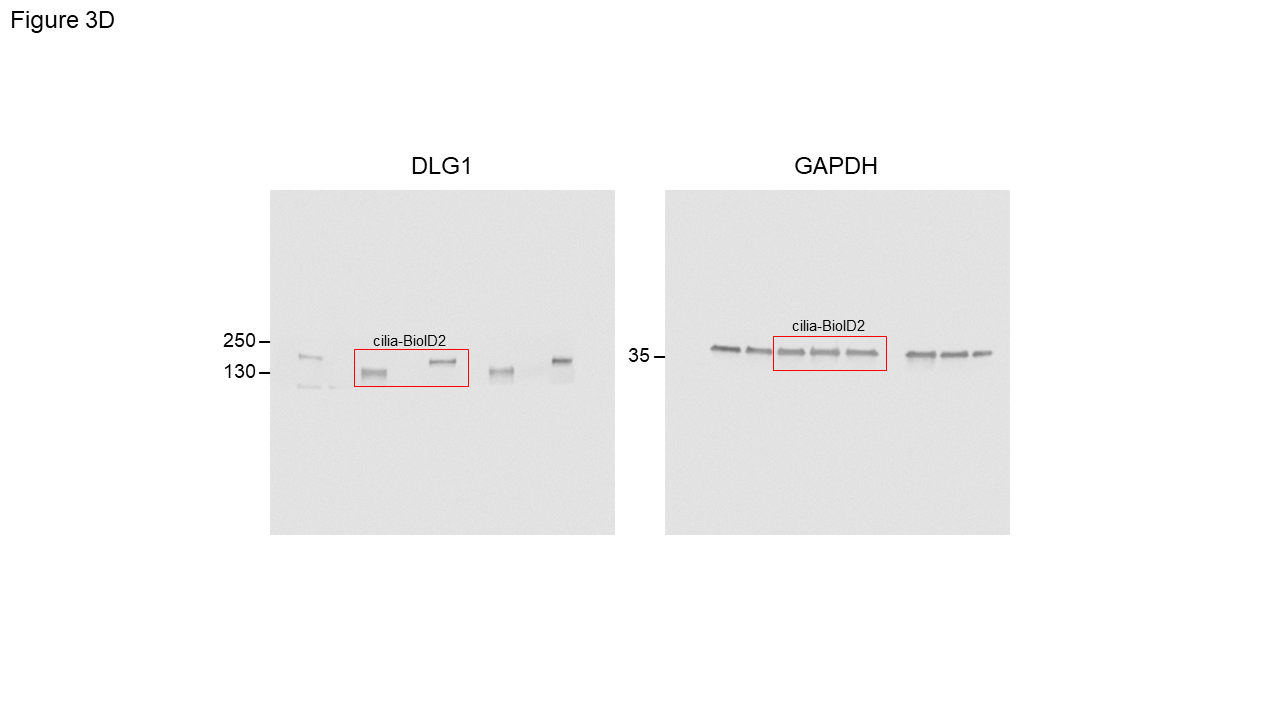

Supplement: Supplementary file 5 — Source data Fig. 3 [file 44319_2024_170_MOESM5_ESM.zip › Figure 3 Source Data/Panel 3D/figure3D_labeling.tif]

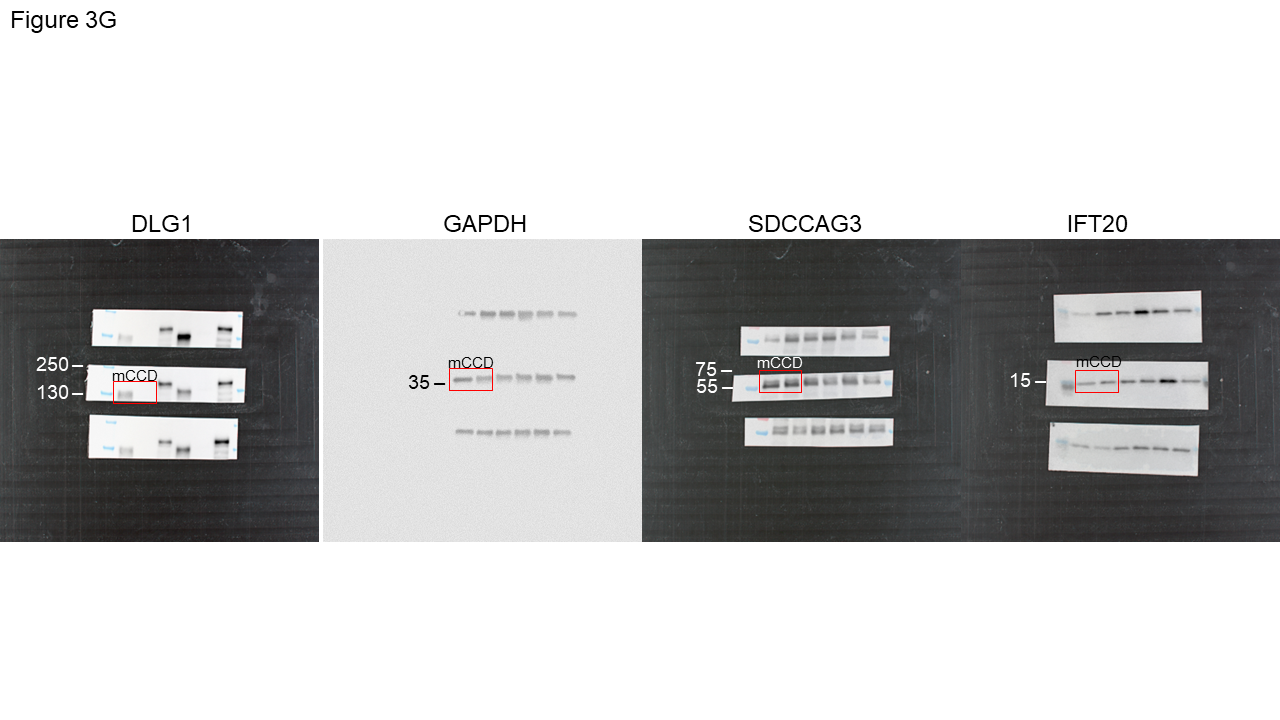

Supplement: Supplementary file 5 — Source data Fig. 3 [file 44319_2024_170_MOESM5_ESM.zip › Figure 3 Source Data/Panel 3G/figure3G_labeling.tif]

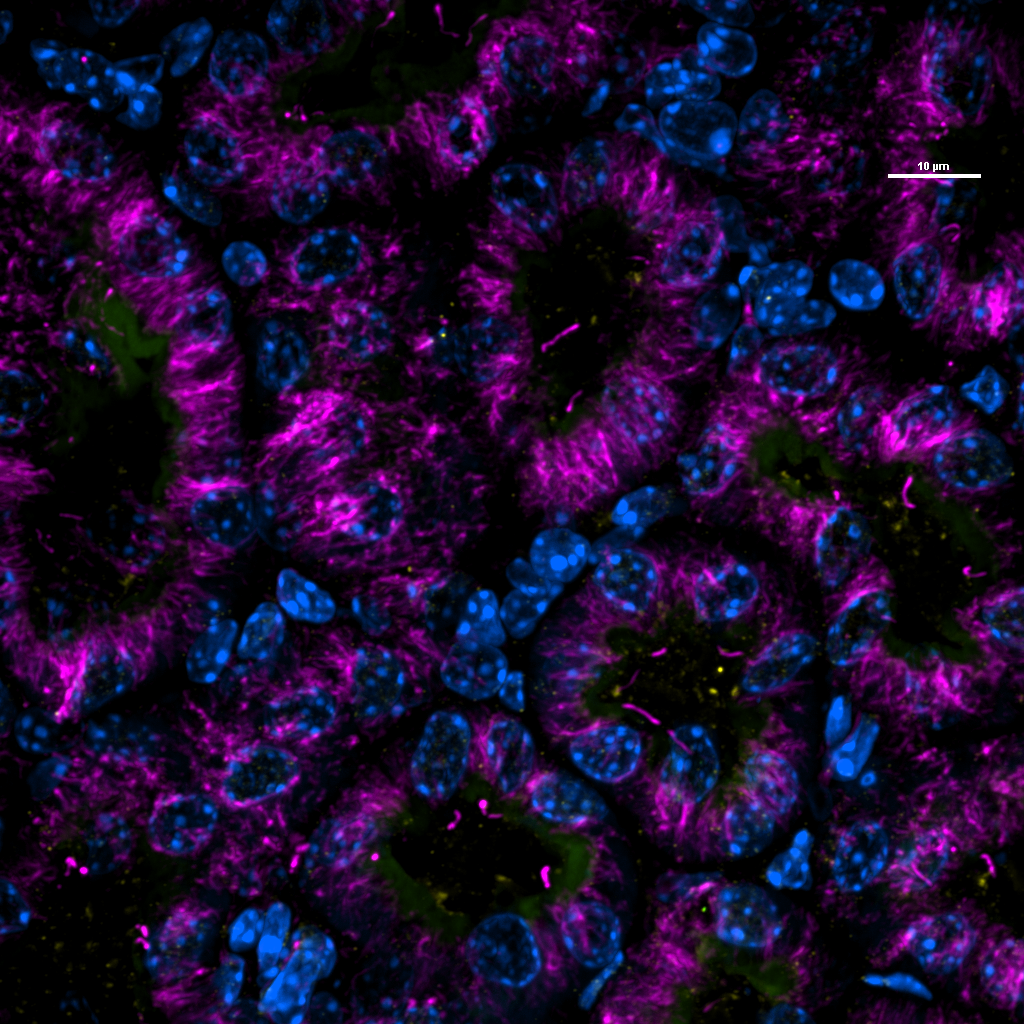

Supplement: Supplementary file 6 — Source data Fig. 4 [file 44319_2024_170_MOESM6_ESM.zip › Figure 4 Source Data/Panel 4A/Pax3Cre-Dlg1FF-SDCCAG3-60x-RGB.tif]

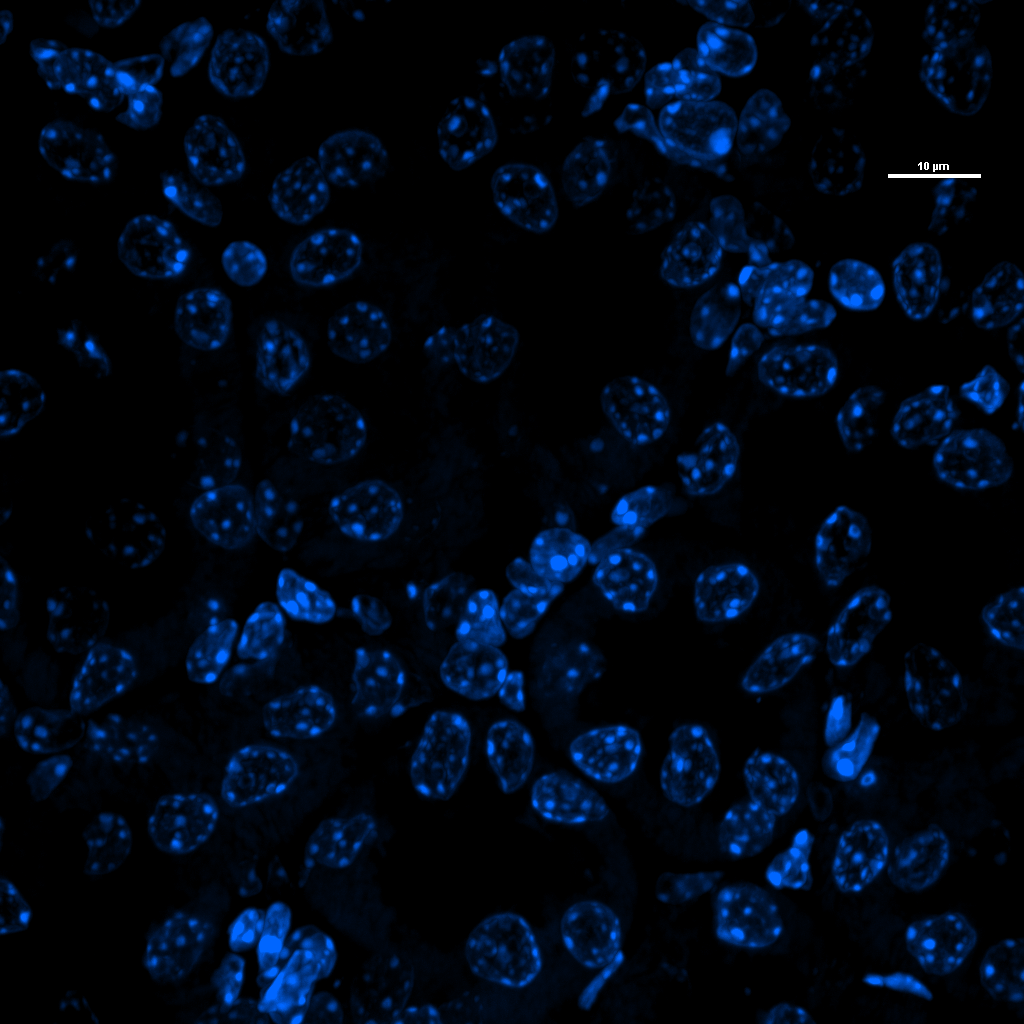

Supplement: Supplementary file 6 — Source data Fig. 4 [file 44319_2024_170_MOESM6_ESM.zip › Figure 4 Source Data/Panel 4A/Pax3Cre-Dlg1FF-SDCCAG3-60x-RGB_405-SD.tif]

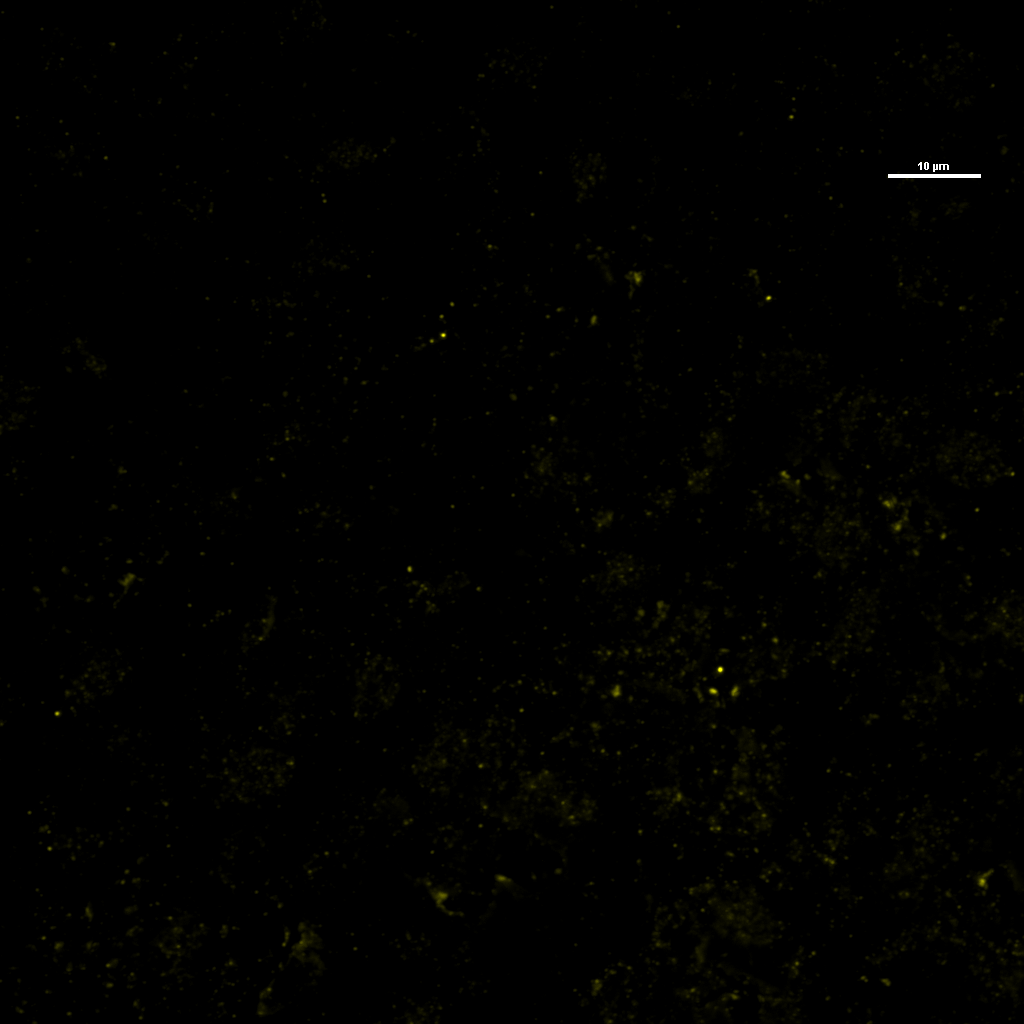

Supplement: Supplementary file 6 — Source data Fig. 4 [file 44319_2024_170_MOESM6_ESM.zip › Figure 4 Source Data/Panel 4A/Pax3Cre-Dlg1FF-SDCCAG3-60x-RGB_561-SD.tif]

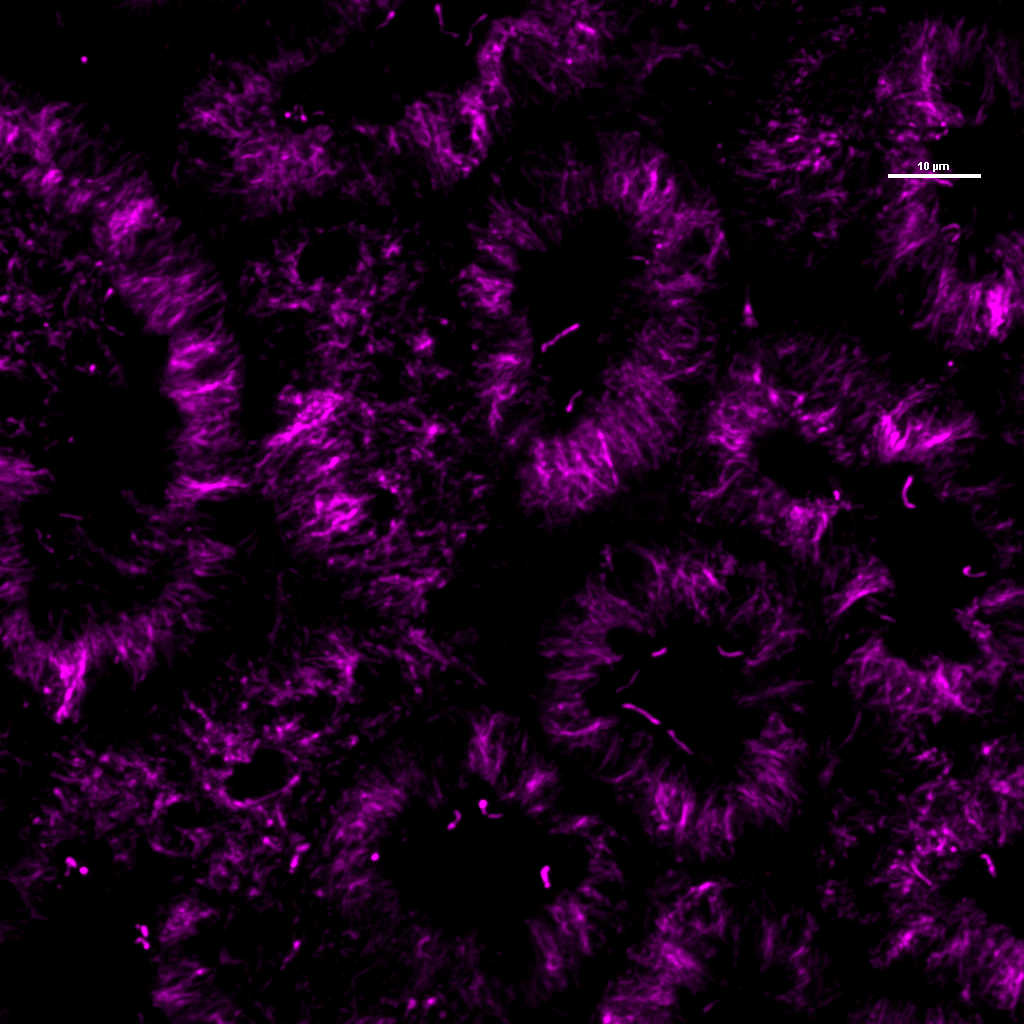

Supplement: Supplementary file 6 — Source data Fig. 4 [file 44319_2024_170_MOESM6_ESM.zip › Figure 4 Source Data/Panel 4A/Pax3Cre-Dlg1FF-SDCCAG3-60x-RGB_640-SD.tif]

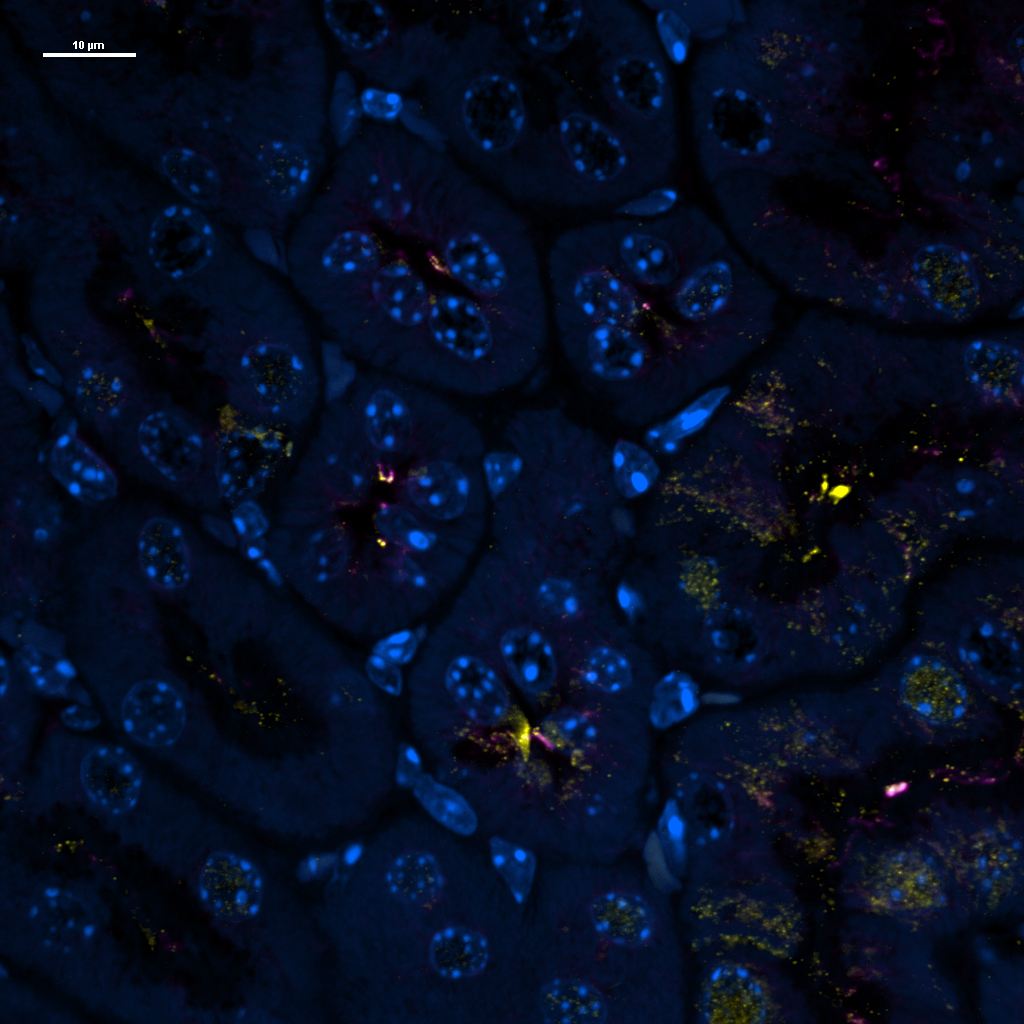

Supplement: Supplementary file 6 — Source data Fig. 4 [file 44319_2024_170_MOESM6_ESM.zip › Figure 4 Source Data/Panel 4A/Wildtype-SDCCAG3-60x-RGB.tif]

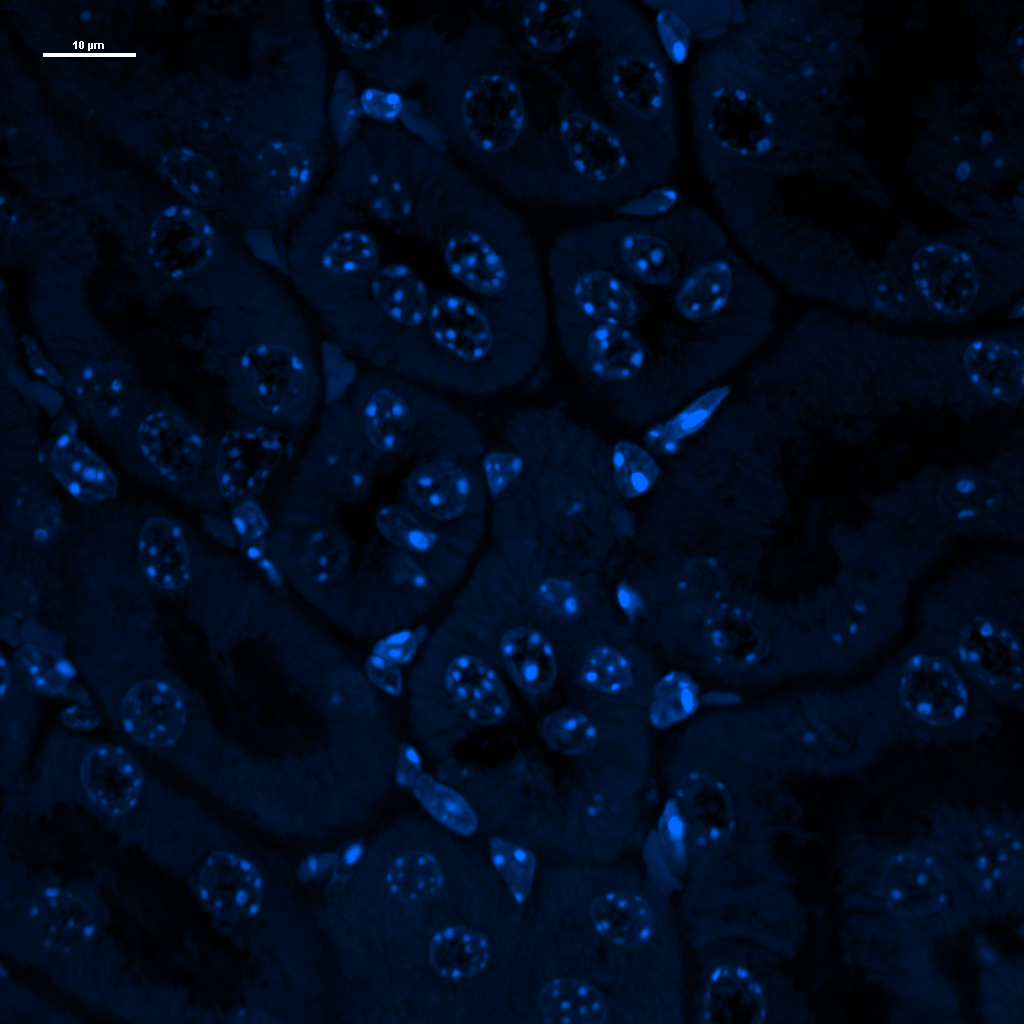

Supplement: Supplementary file 6 — Source data Fig. 4 [file 44319_2024_170_MOESM6_ESM.zip › Figure 4 Source Data/Panel 4A/Wildtype-SDCCAG3-60x-RGB_405-SD.tif]

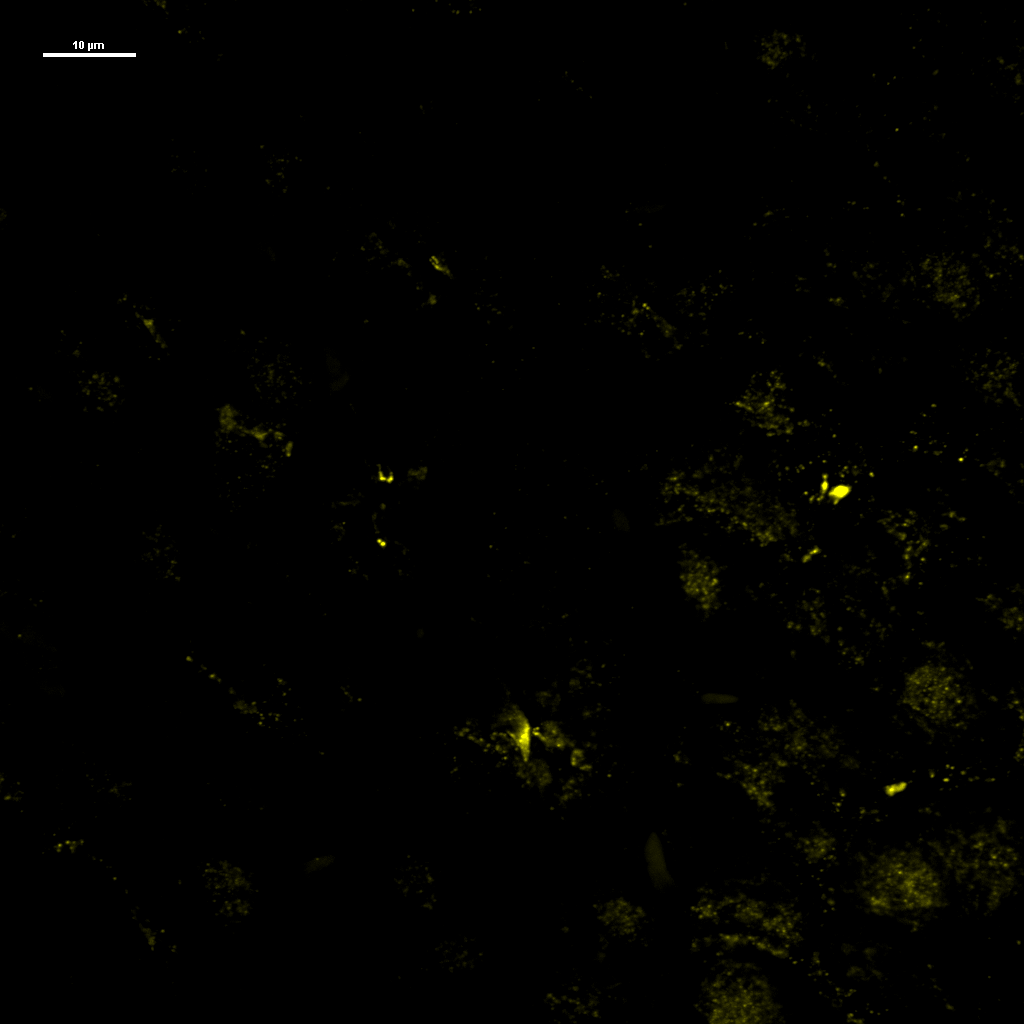

Supplement: Supplementary file 6 — Source data Fig. 4 [file 44319_2024_170_MOESM6_ESM.zip › Figure 4 Source Data/Panel 4A/Wildtype-SDCCAG3-60x-RGB_561-SD.tif]

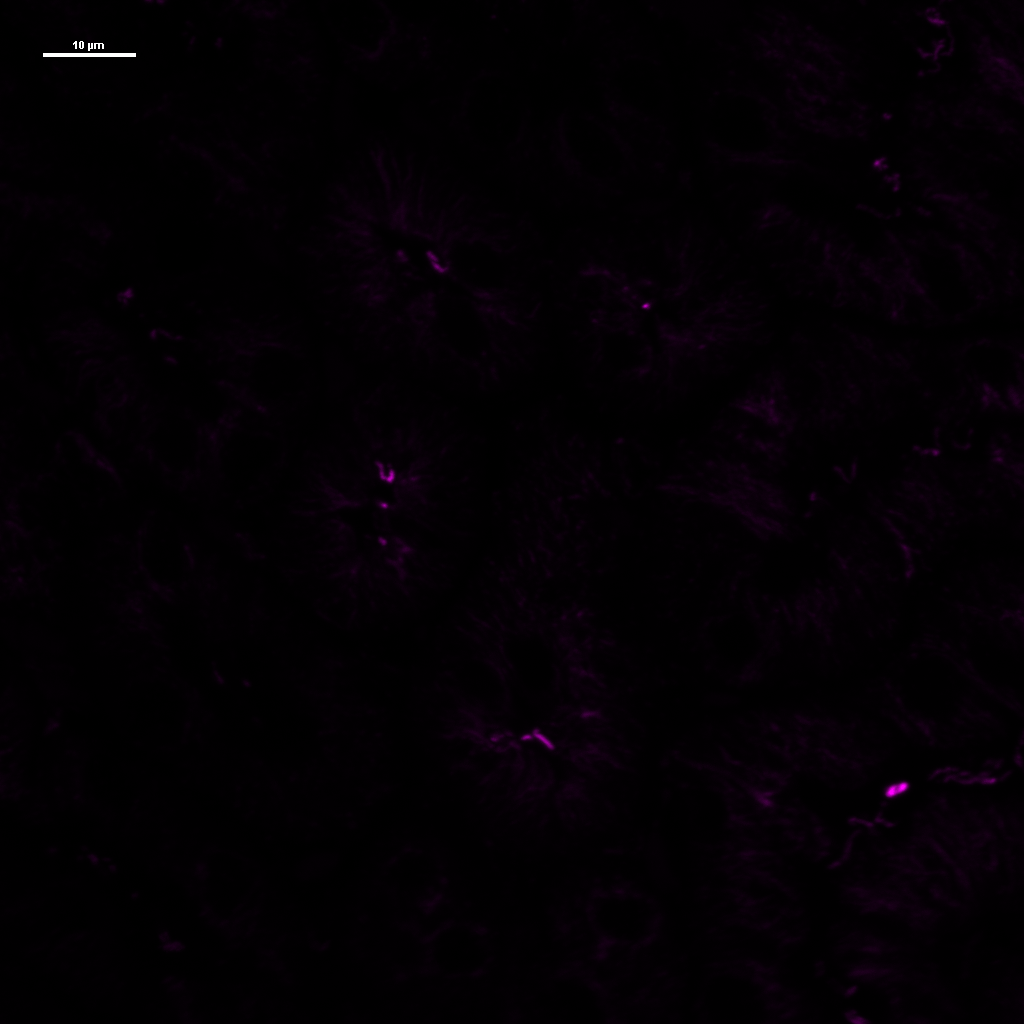

Supplement: Supplementary file 6 — Source data Fig. 4 [file 44319_2024_170_MOESM6_ESM.zip › Figure 4 Source Data/Panel 4A/Wildtype-SDCCAG3-60x-RGB_640-SD.tif]

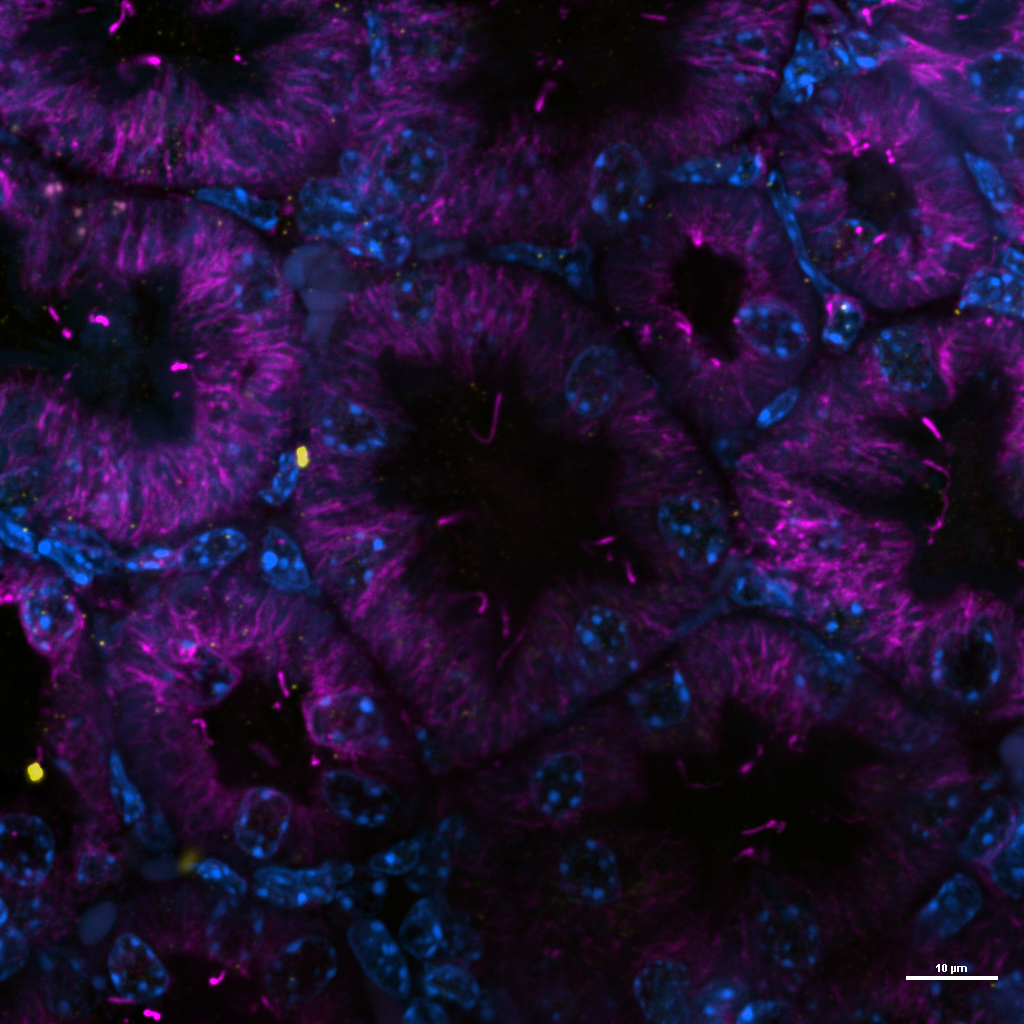

Supplement: Supplementary file 6 — Source data Fig. 4 [file 44319_2024_170_MOESM6_ESM.zip › Figure 4 Source Data/Panel 4B/Pax3Cre-Dlg1FF-IFT20-60x-RGB.tif]

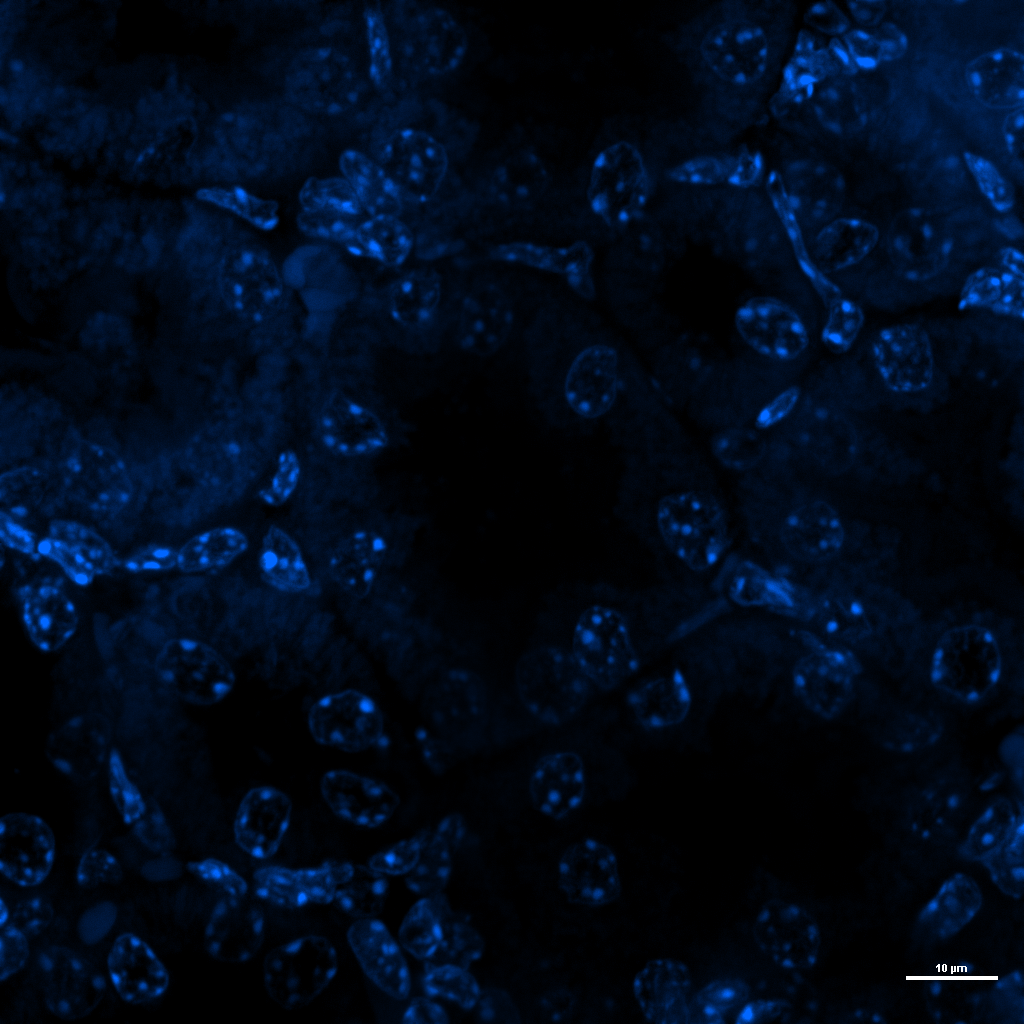

Supplement: Supplementary file 6 — Source data Fig. 4 [file 44319_2024_170_MOESM6_ESM.zip › Figure 4 Source Data/Panel 4B/Pax3Cre-Dlg1FF-IFT20-60x-RGB_405-SD.tif]

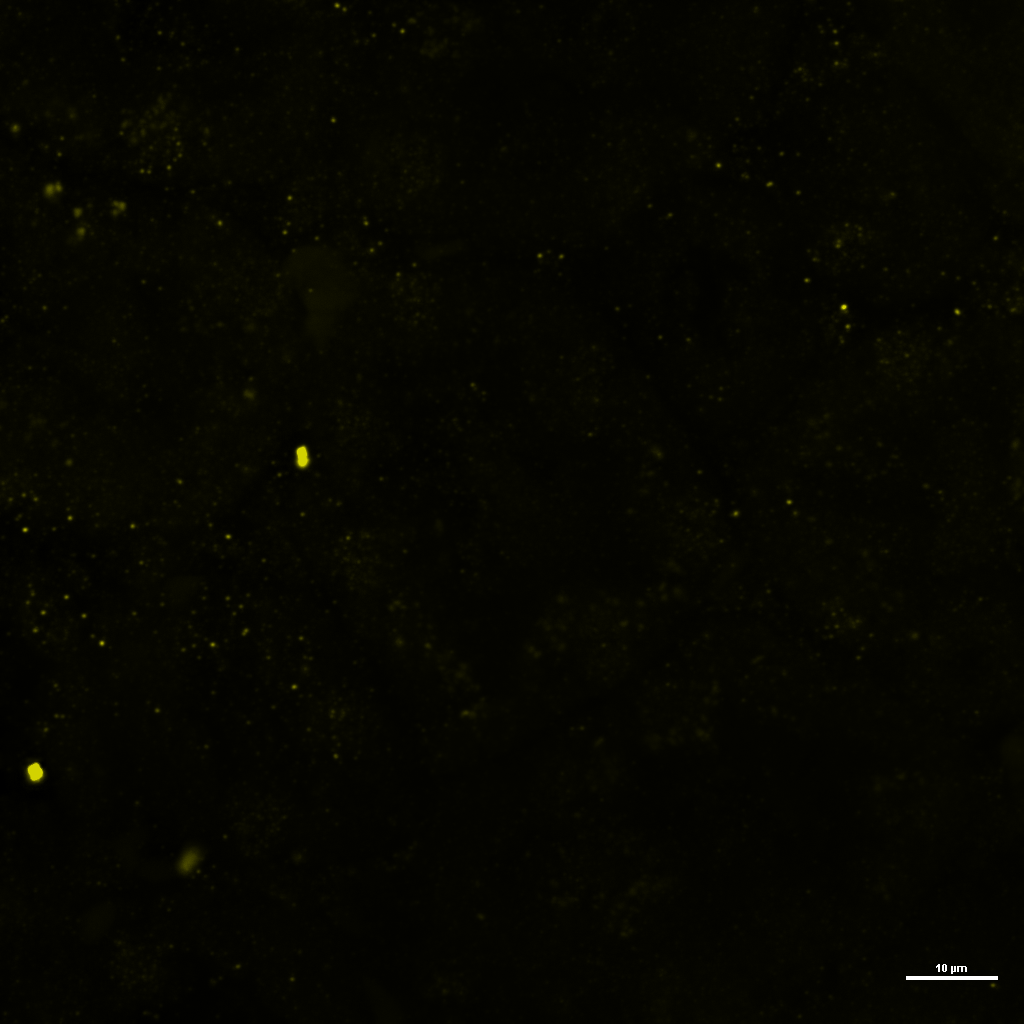

Supplement: Supplementary file 6 — Source data Fig. 4 [file 44319_2024_170_MOESM6_ESM.zip › Figure 4 Source Data/Panel 4B/Pax3Cre-Dlg1FF-IFT20-60x-RGB_561-SD.tif]

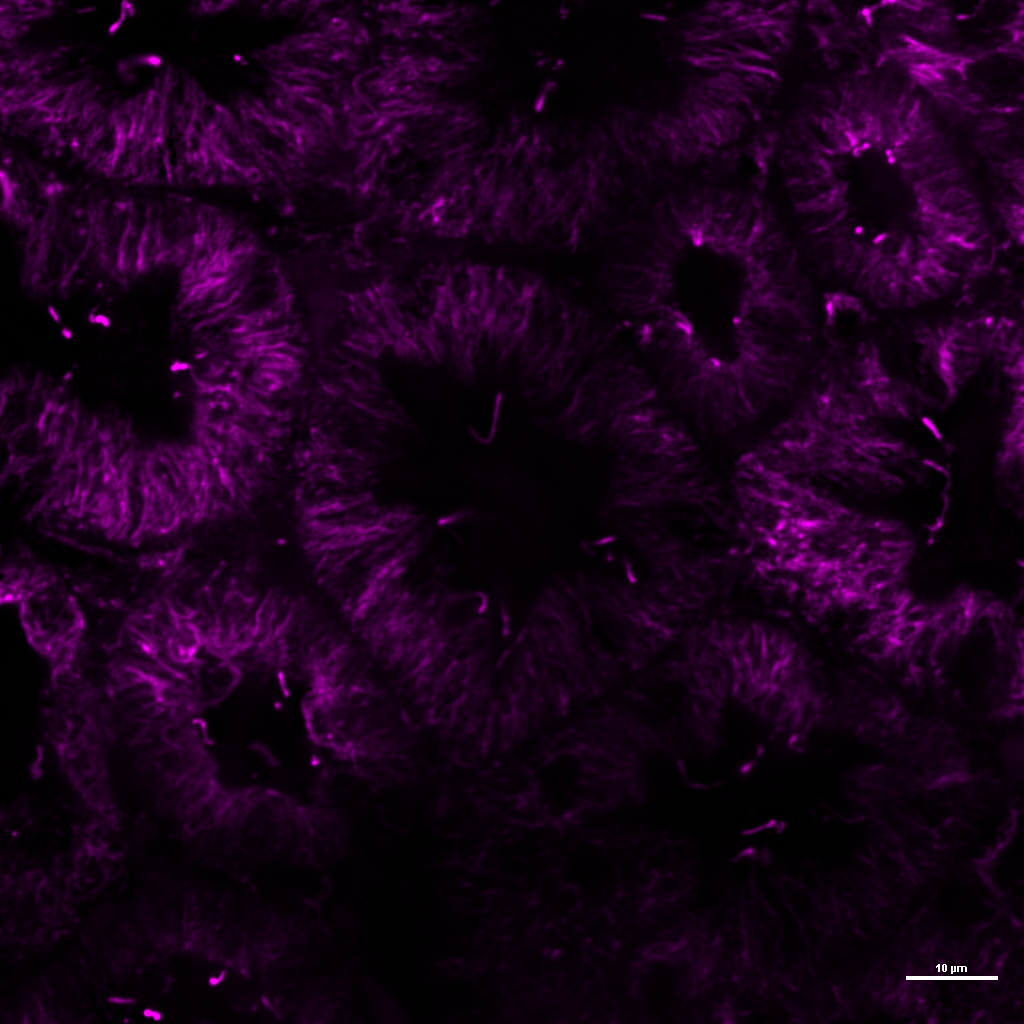

Supplement: Supplementary file 6 — Source data Fig. 4 [file 44319_2024_170_MOESM6_ESM.zip › Figure 4 Source Data/Panel 4B/Pax3Cre-Dlg1FF-IFT20-60x-RGB_640-SD.tif]

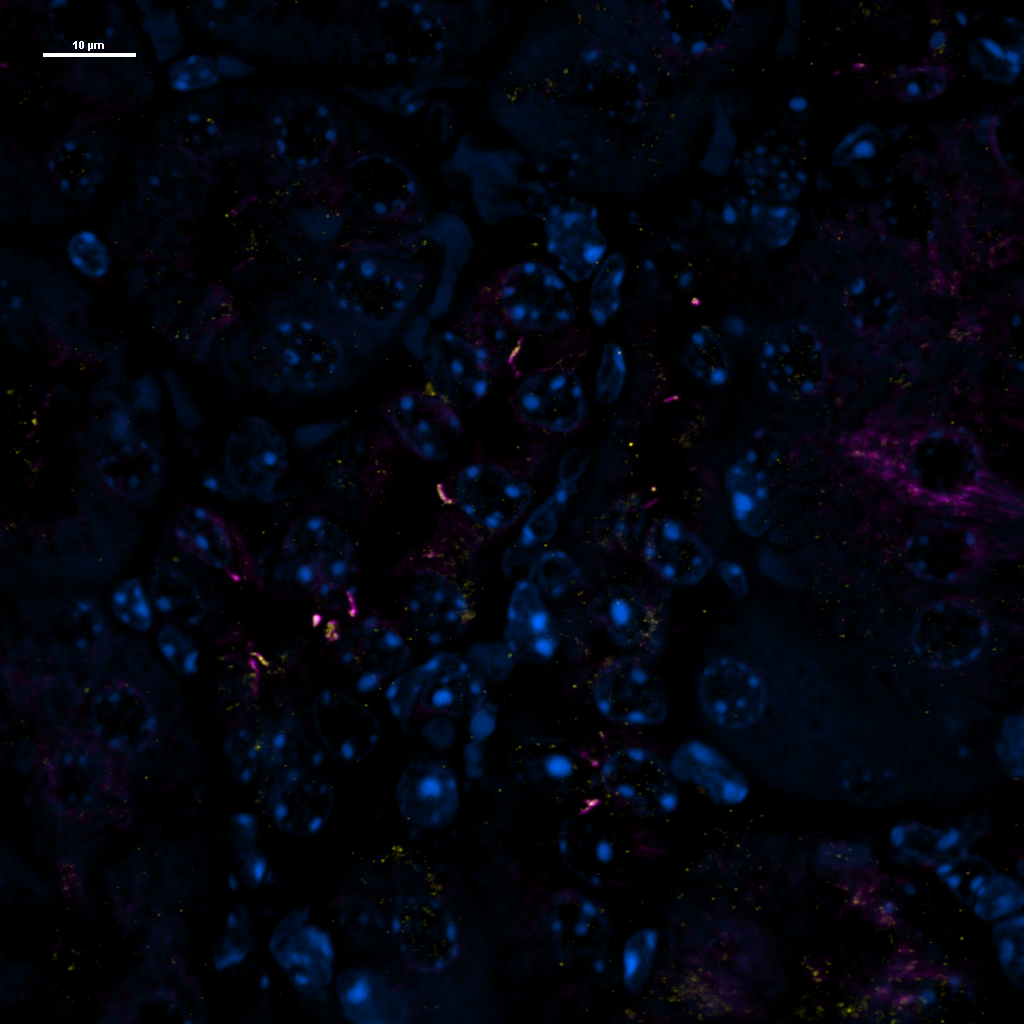

Supplement: Supplementary file 6 — Source data Fig. 4 [file 44319_2024_170_MOESM6_ESM.zip › Figure 4 Source Data/Panel 4B/Wildtype-IFT20-60x-RGB.tif]

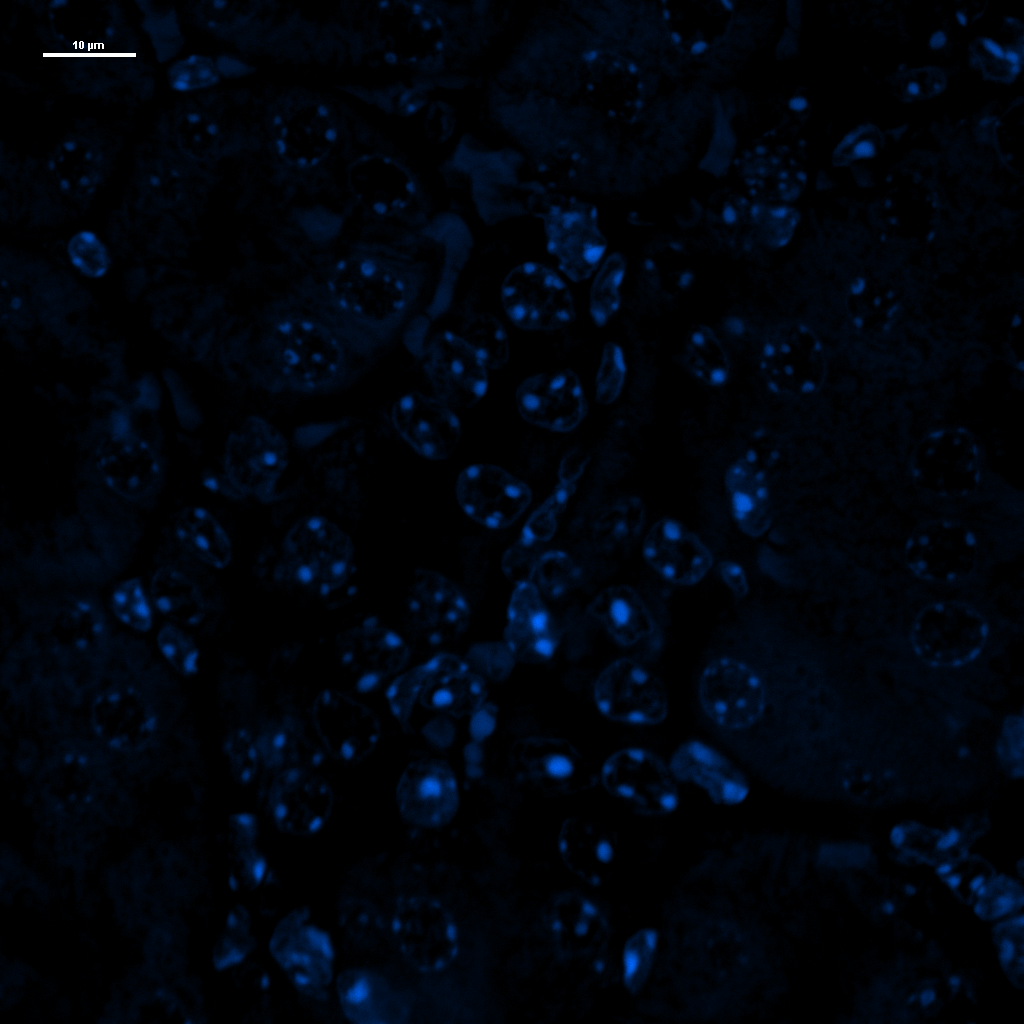

Supplement: Supplementary file 6 — Source data Fig. 4 [file 44319_2024_170_MOESM6_ESM.zip › Figure 4 Source Data/Panel 4B/Wildtype-IFT20-60x-RGB_405-SD.tif]

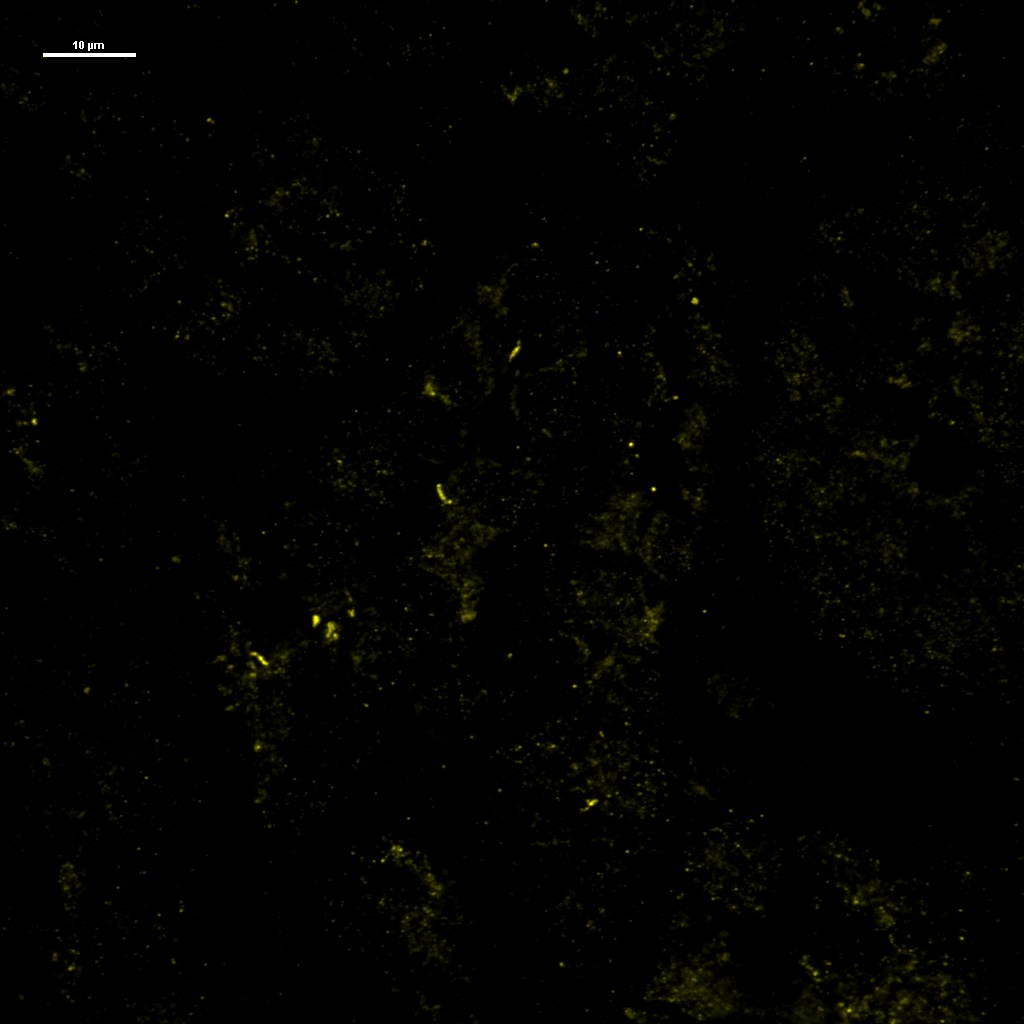

Supplement: Supplementary file 6 — Source data Fig. 4 [file 44319_2024_170_MOESM6_ESM.zip › Figure 4 Source Data/Panel 4B/Wildtype-IFT20-60x-RGB_561-SD.tif]

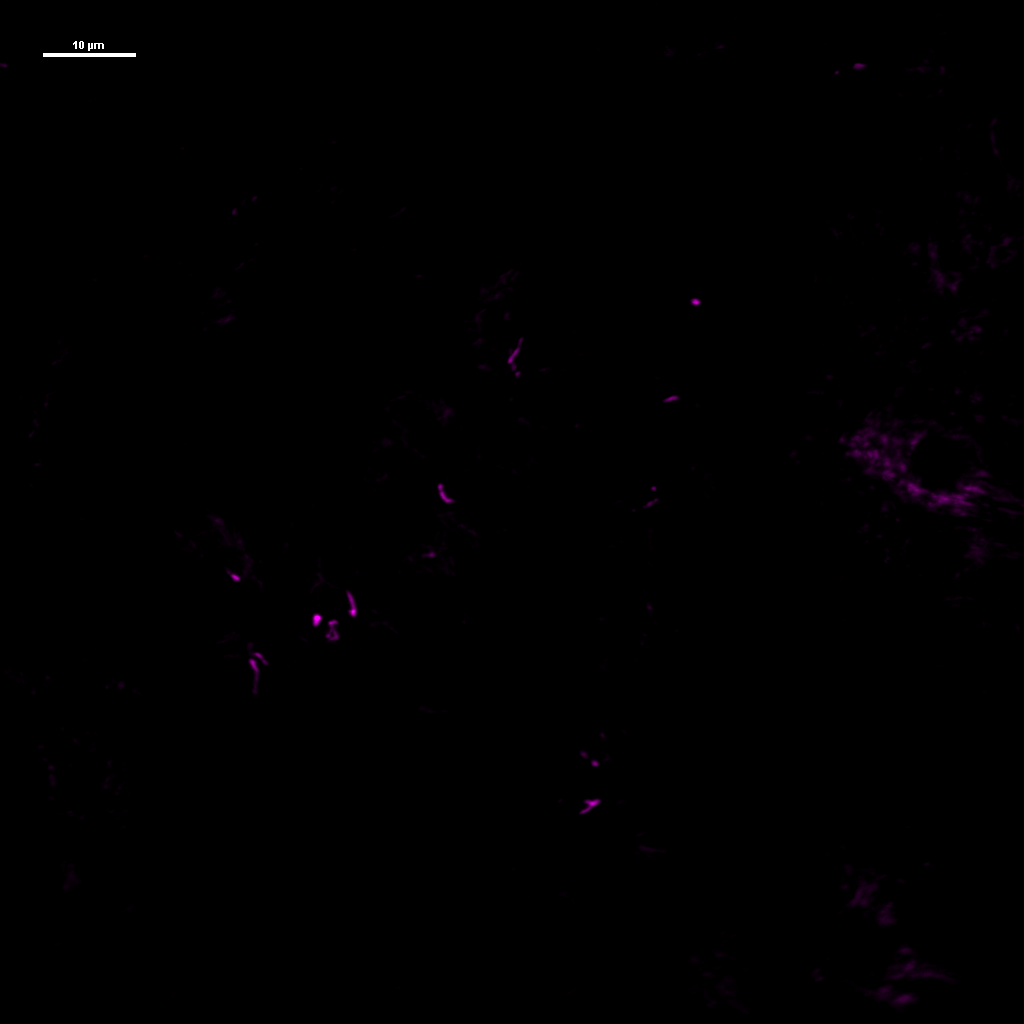

Supplement: Supplementary file 6 — Source data Fig. 4 [file 44319_2024_170_MOESM6_ESM.zip › Figure 4 Source Data/Panel 4B/Wildtype-IFT20-60x-RGB_640-SD.tif]

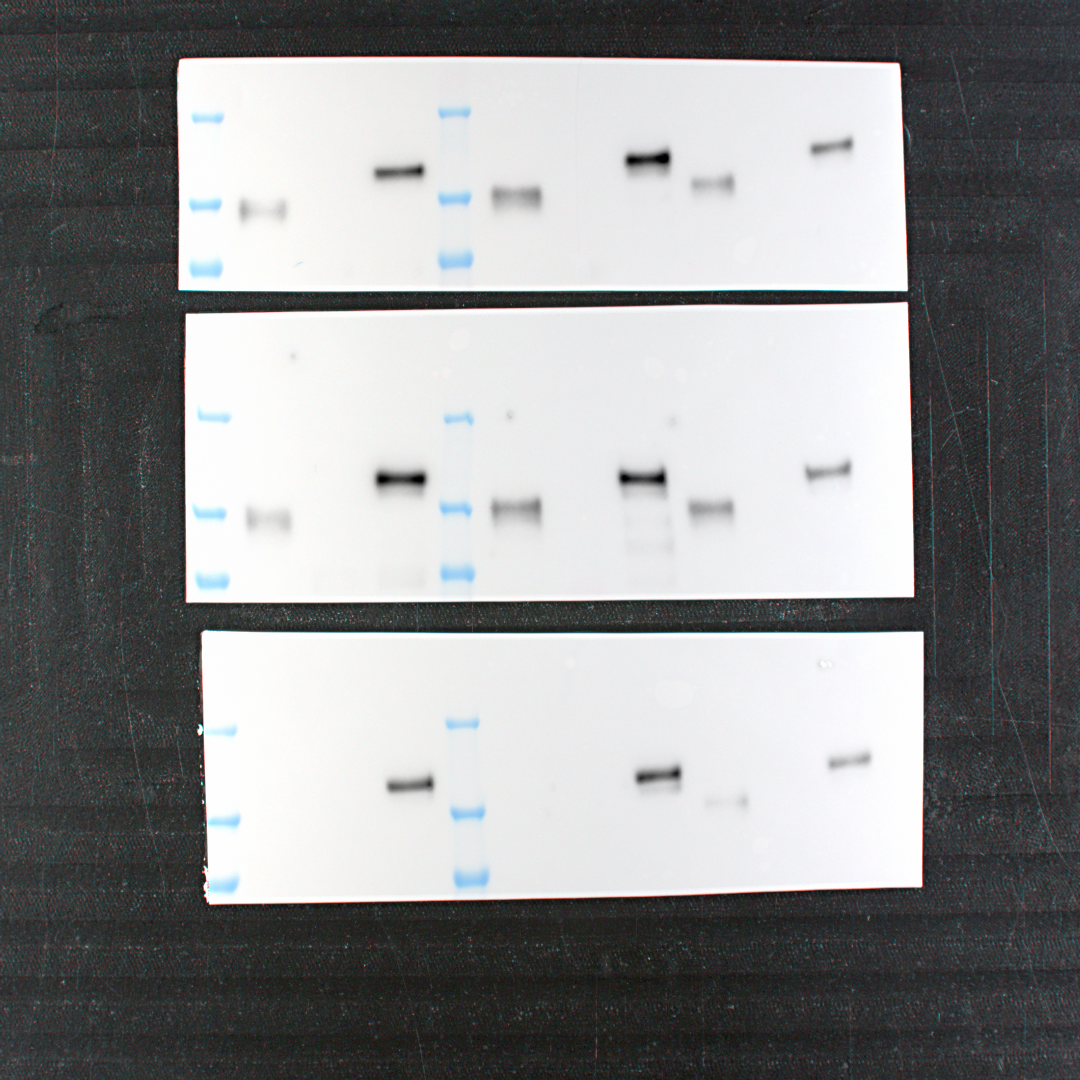

Supplement: Supplementary file 8 — Source data Fig. 6 [file 44319_2024_170_MOESM8_ESM.zip › Figure 6 Source Data/Panel 6B/DLG1_with-ladder.Tif]

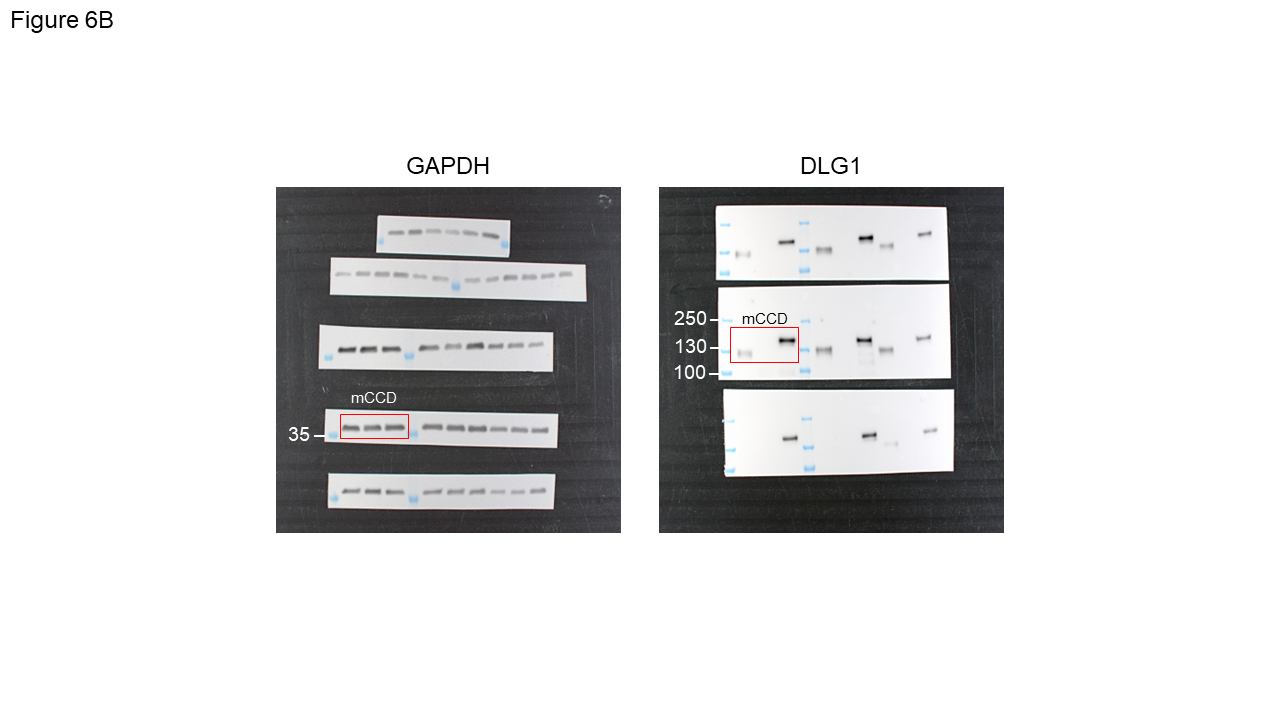

Supplement: Supplementary file 8 — Source data Fig. 6 [file 44319_2024_170_MOESM8_ESM.zip › Figure 6 Source Data/Panel 6B/figure6B_labeling.tif]

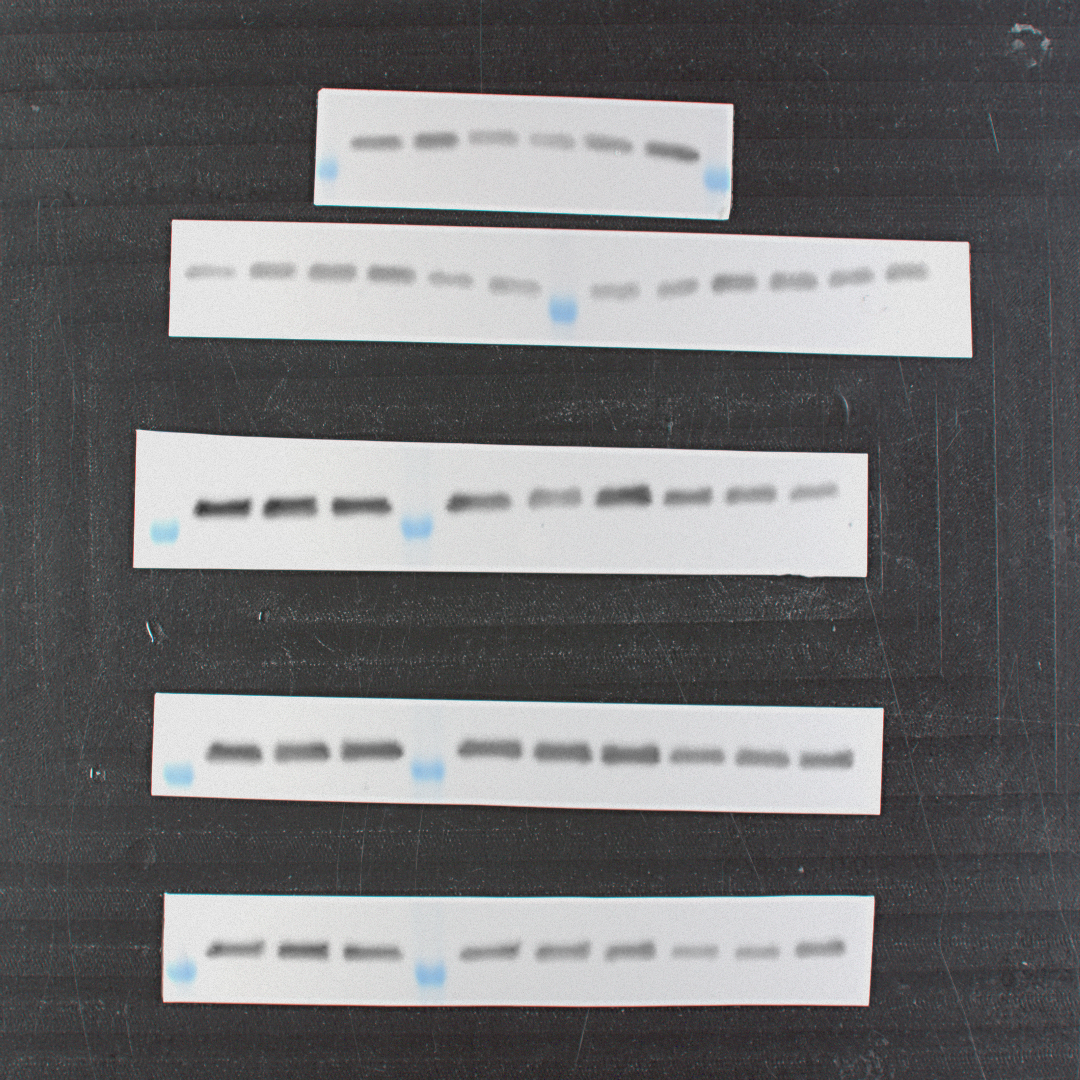

Supplement: Supplementary file 8 — Source data Fig. 6 [file 44319_2024_170_MOESM8_ESM.zip › Figure 6 Source Data/Panel 6B/GAPDH_with-ladder.Tif]

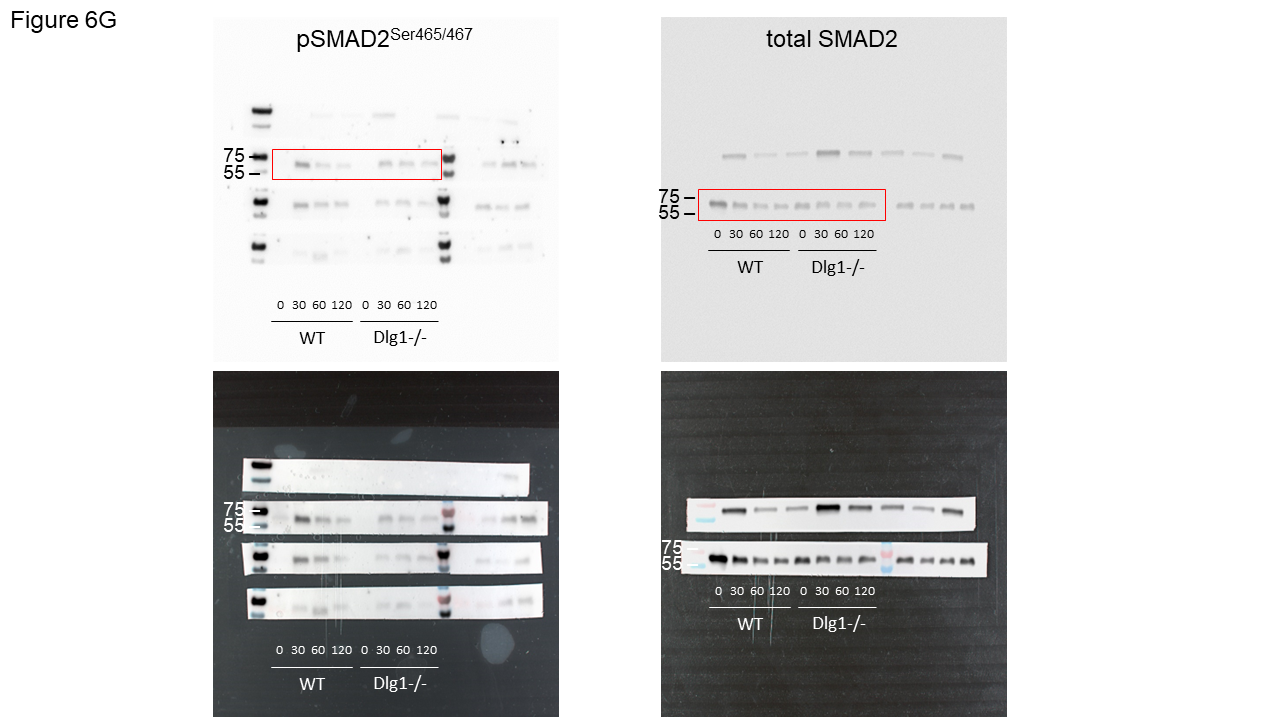

Supplement: Supplementary file 8 — Source data Fig. 6 [file 44319_2024_170_MOESM8_ESM.zip › Figure 6 Source Data/Panel 6H/figure6G_labeling.tif]

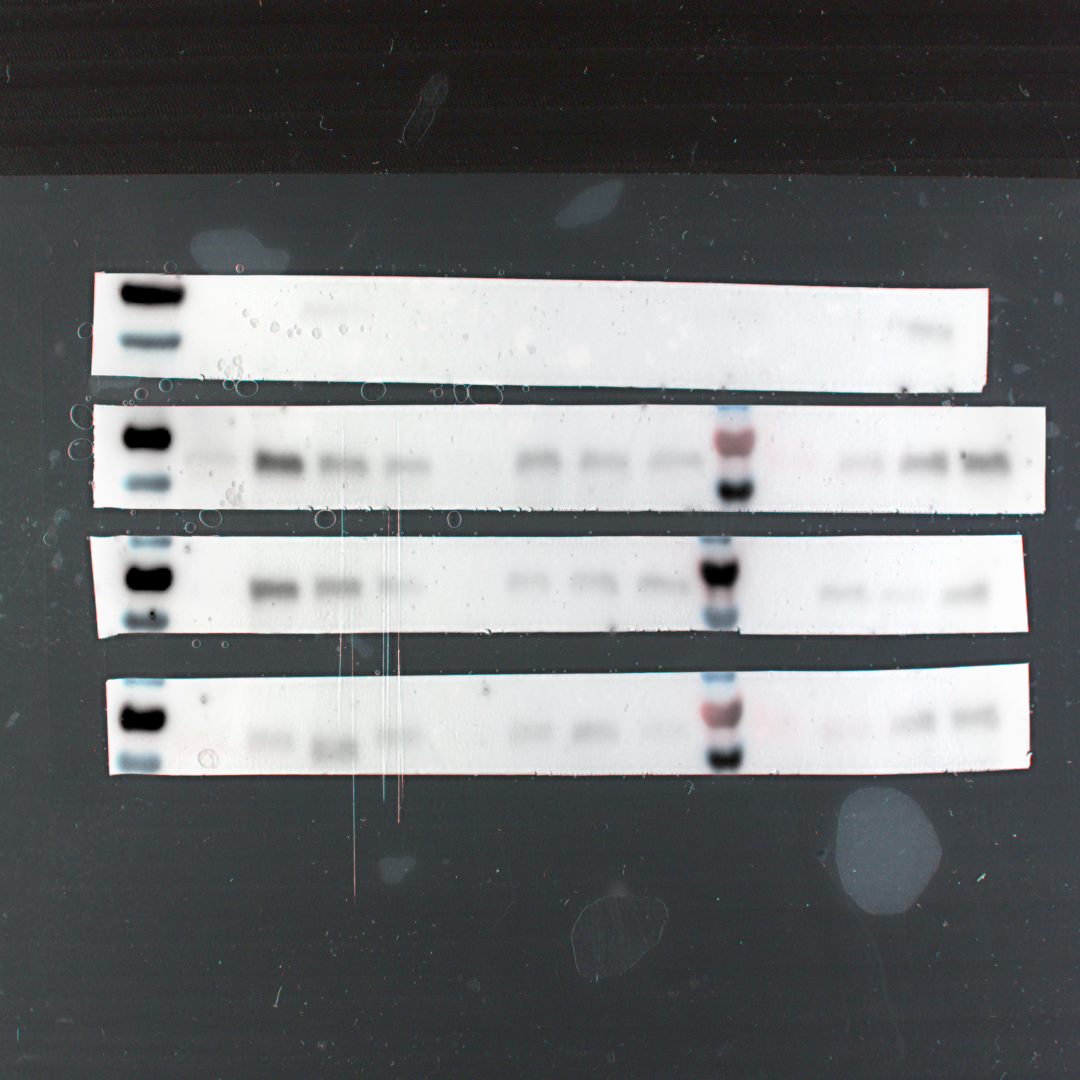

Supplement: Supplementary file 8 — Source data Fig. 6 [file 44319_2024_170_MOESM8_ESM.zip › Figure 6 Source Data/Panel 6H/pSMAD2_with-ladder.Tif]

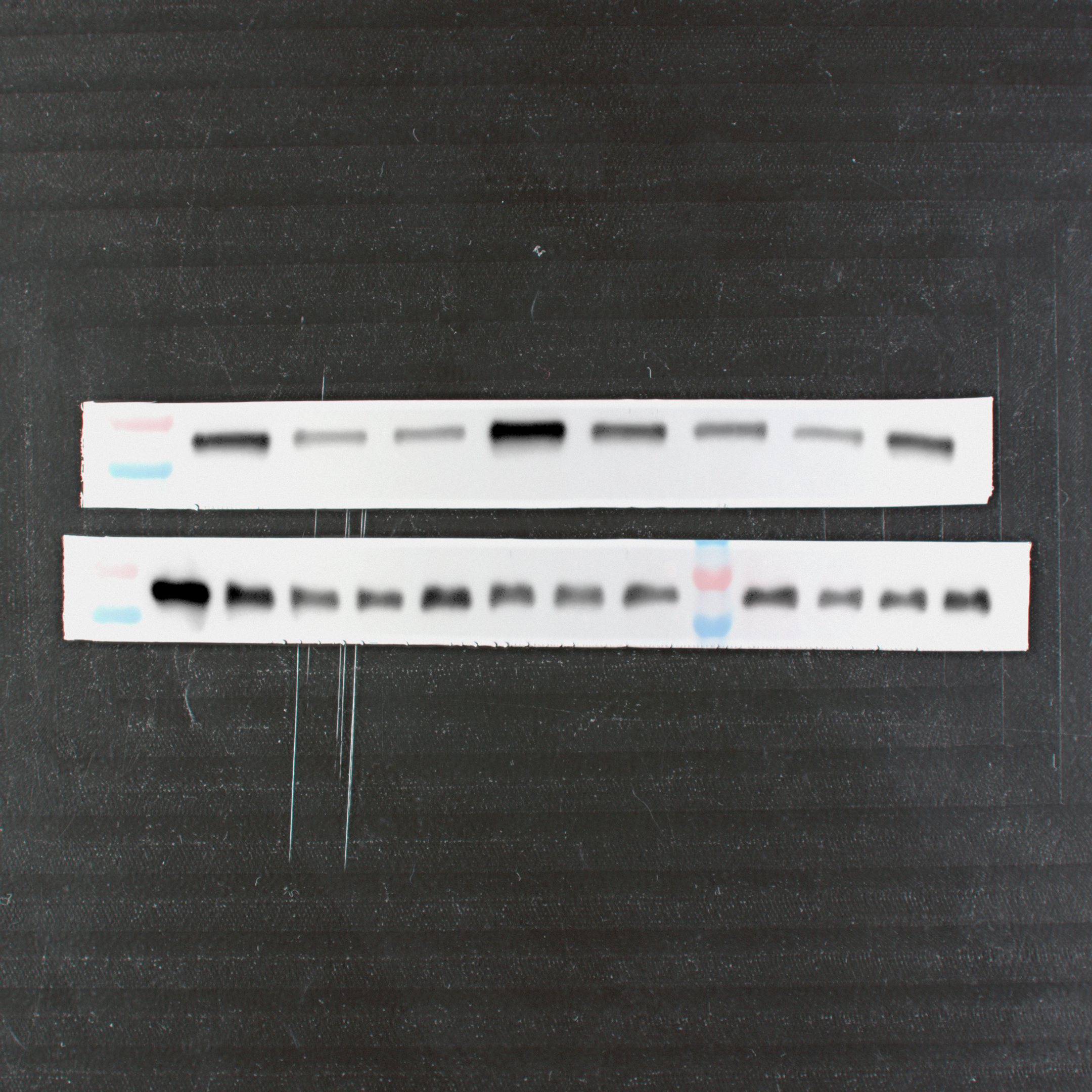

Supplement: Supplementary file 8 — Source data Fig. 6 [file 44319_2024_170_MOESM8_ESM.zip › Figure 6 Source Data/Panel 6H/SMAD2_with-ladder.Tif]

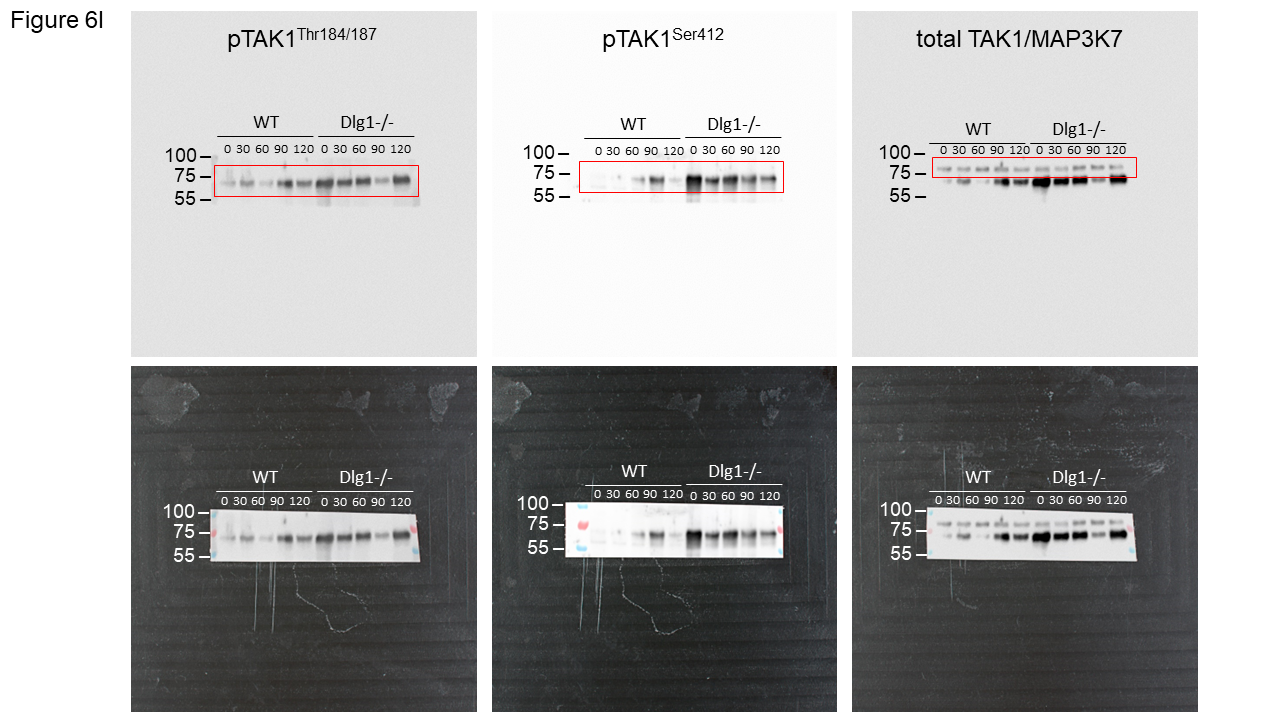

Supplement: Supplementary file 8 — Source data Fig. 6 [file 44319_2024_170_MOESM8_ESM.zip › Figure 6 Source Data/Panel 6J/figure6I_labeling.tif]

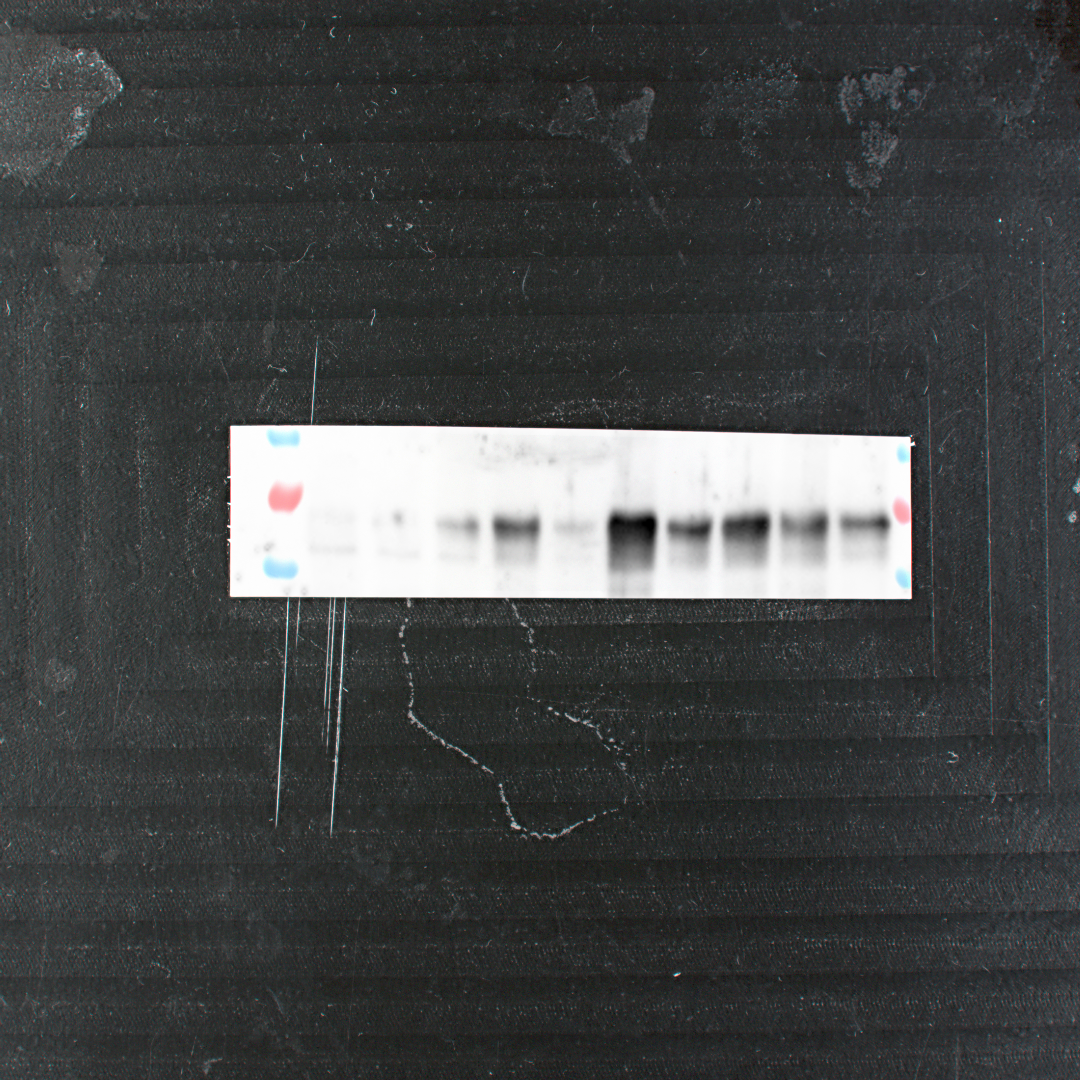

Supplement: Supplementary file 8 — Source data Fig. 6 [file 44319_2024_170_MOESM8_ESM.zip › Figure 6 Source Data/Panel 6J/pTAK1(ser412)_with-ladder.Tif]

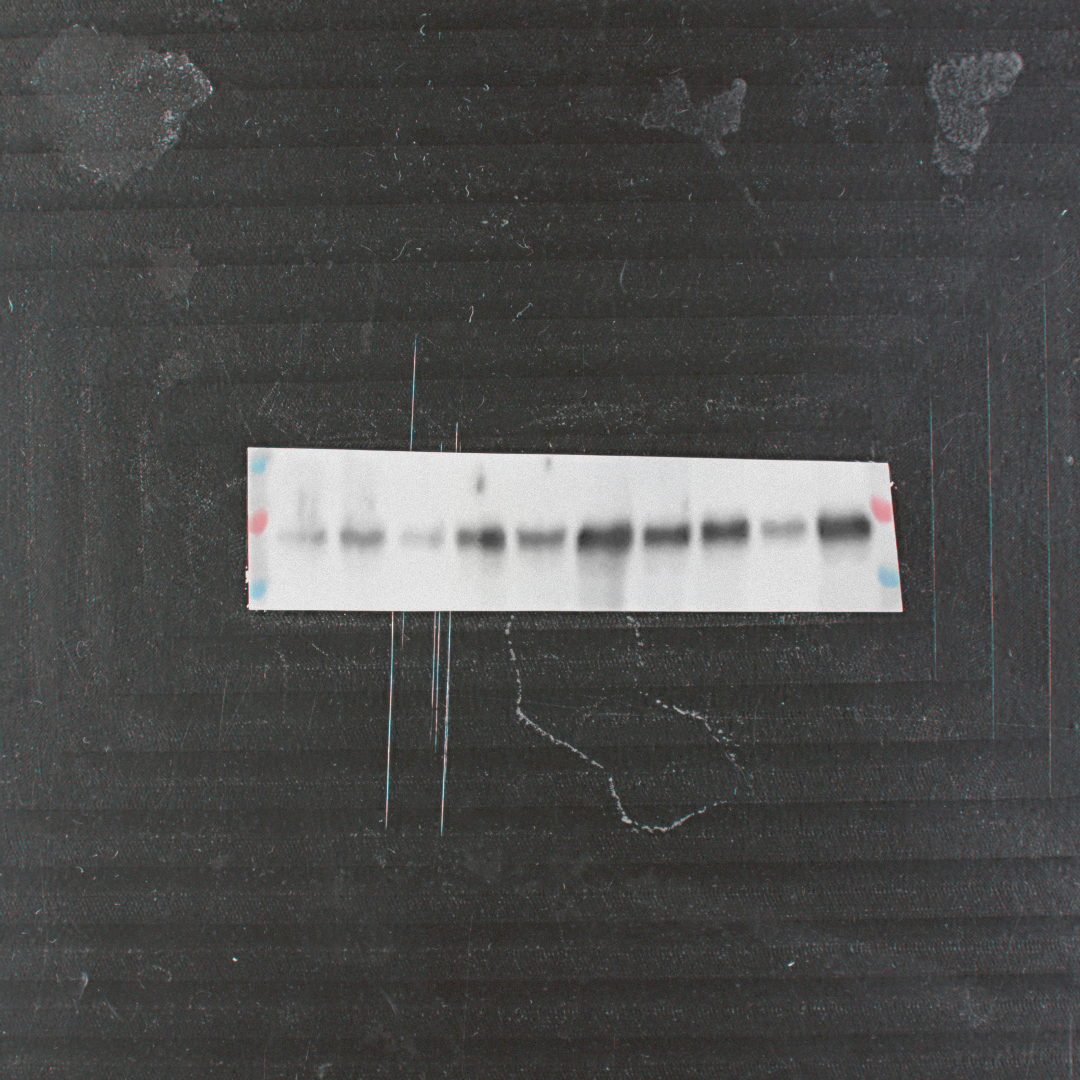

Supplement: Supplementary file 8 — Source data Fig. 6 [file 44319_2024_170_MOESM8_ESM.zip › Figure 6 Source Data/Panel 6J/pTAK1(thr184-187)_with-ladder.Tif]

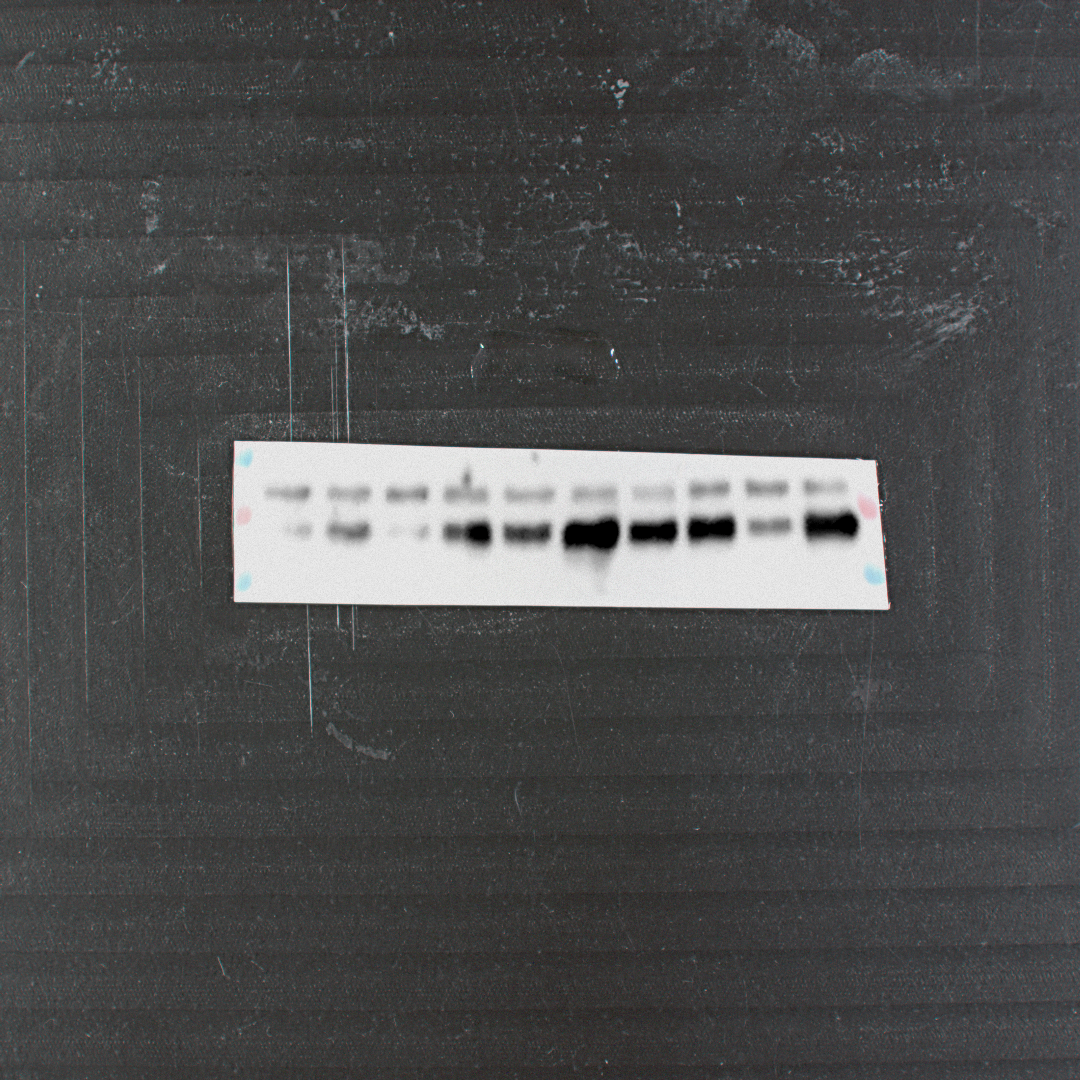

Supplement: Supplementary file 8 — Source data Fig. 6 [file 44319_2024_170_MOESM8_ESM.zip › Figure 6 Source Data/Panel 6J/TAK1_with-ladder.Tif]

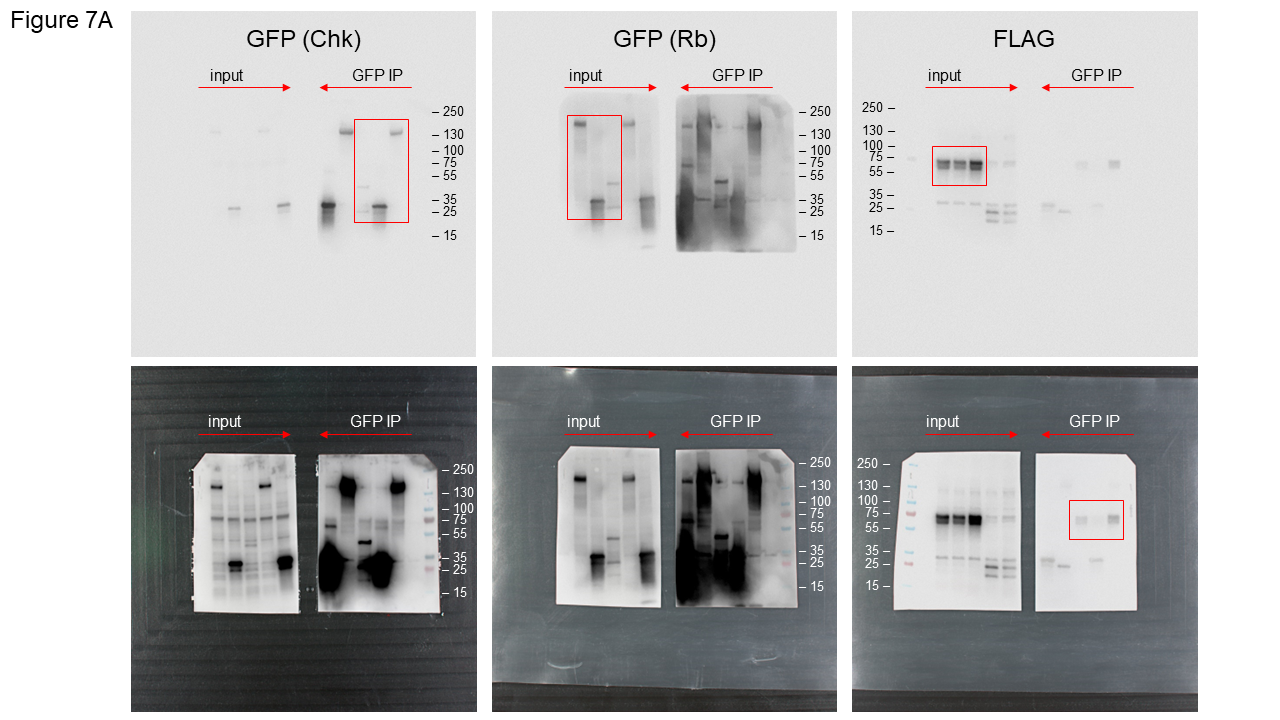

Supplement: Supplementary file 9 — Source data Fig. 7 [file 44319_2024_170_MOESM9_ESM.zip › Figure 7 Source Data/Panel 7A/figure7A_labeling.tif]

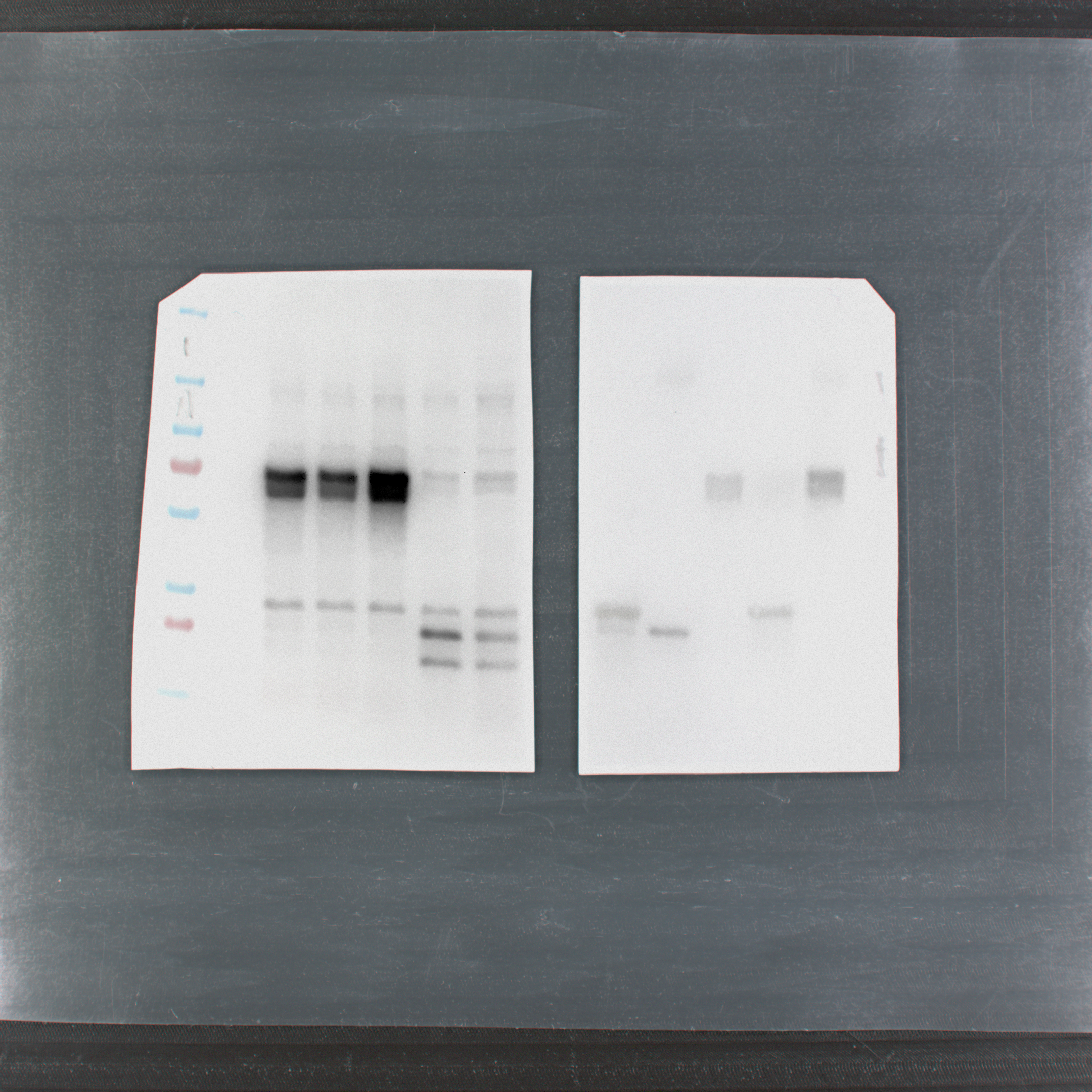

Supplement: Supplementary file 9 — Source data Fig. 7 [file 44319_2024_170_MOESM9_ESM.zip › Figure 7 Source Data/Panel 7A/FLAG-Rb-antibody_with-ladder.Tif]

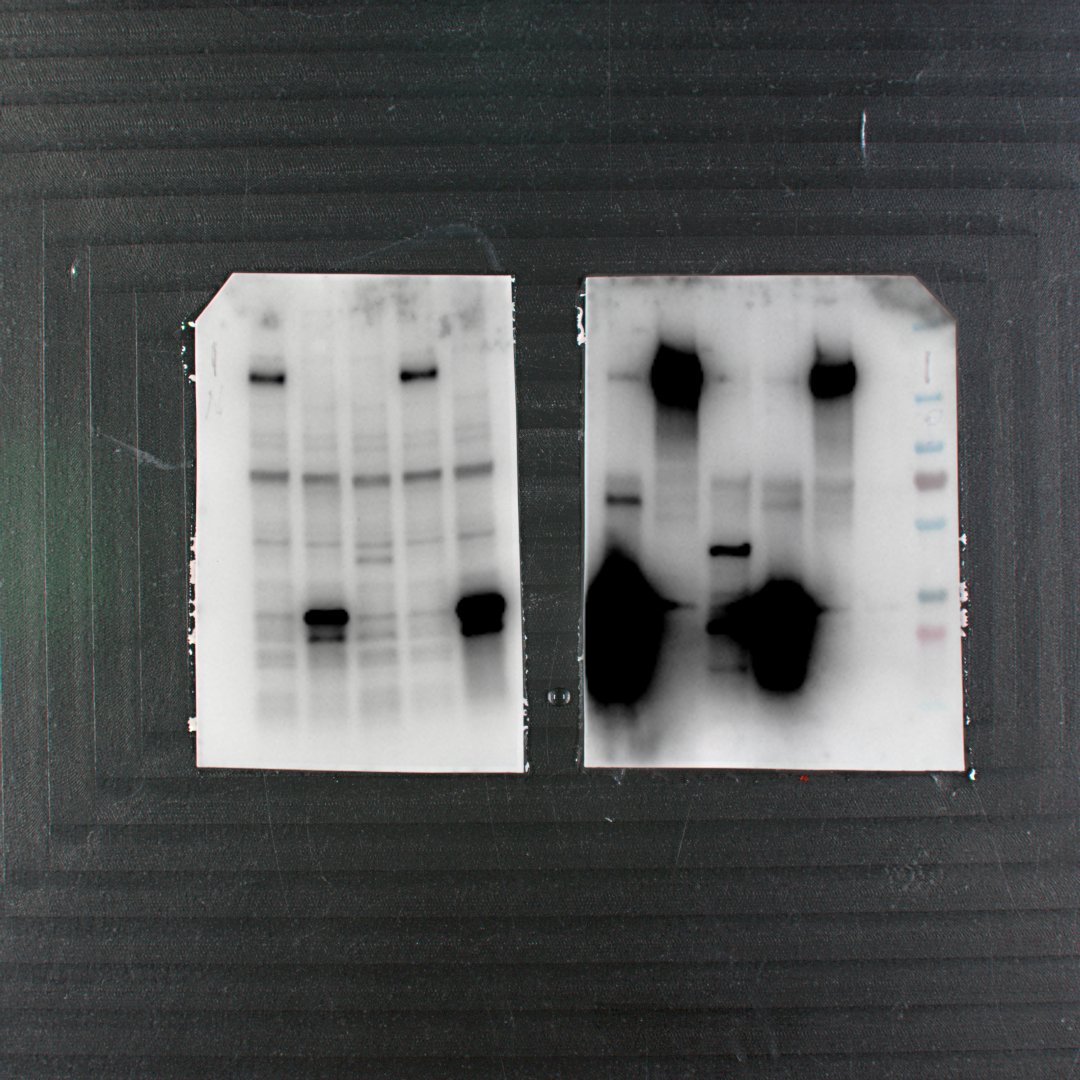

Supplement: Supplementary file 9 — Source data Fig. 7 [file 44319_2024_170_MOESM9_ESM.zip › Figure 7 Source Data/Panel 7A/GFP-Chk-antibody_with-ladder.Tif]

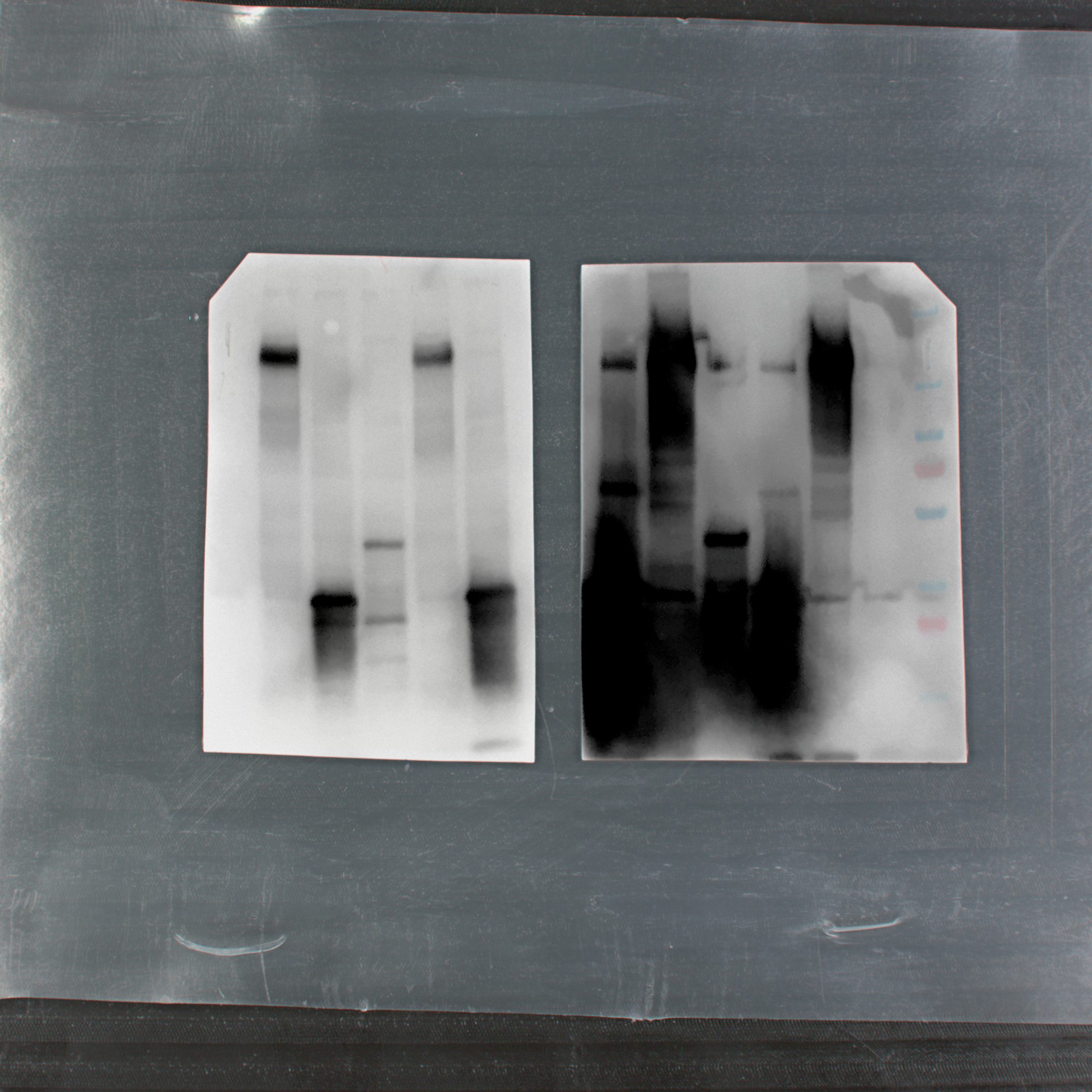

Supplement: Supplementary file 9 — Source data Fig. 7 [file 44319_2024_170_MOESM9_ESM.zip › Figure 7 Source Data/Panel 7A/GFP-Rb-antibody_with-ladder.Tif]

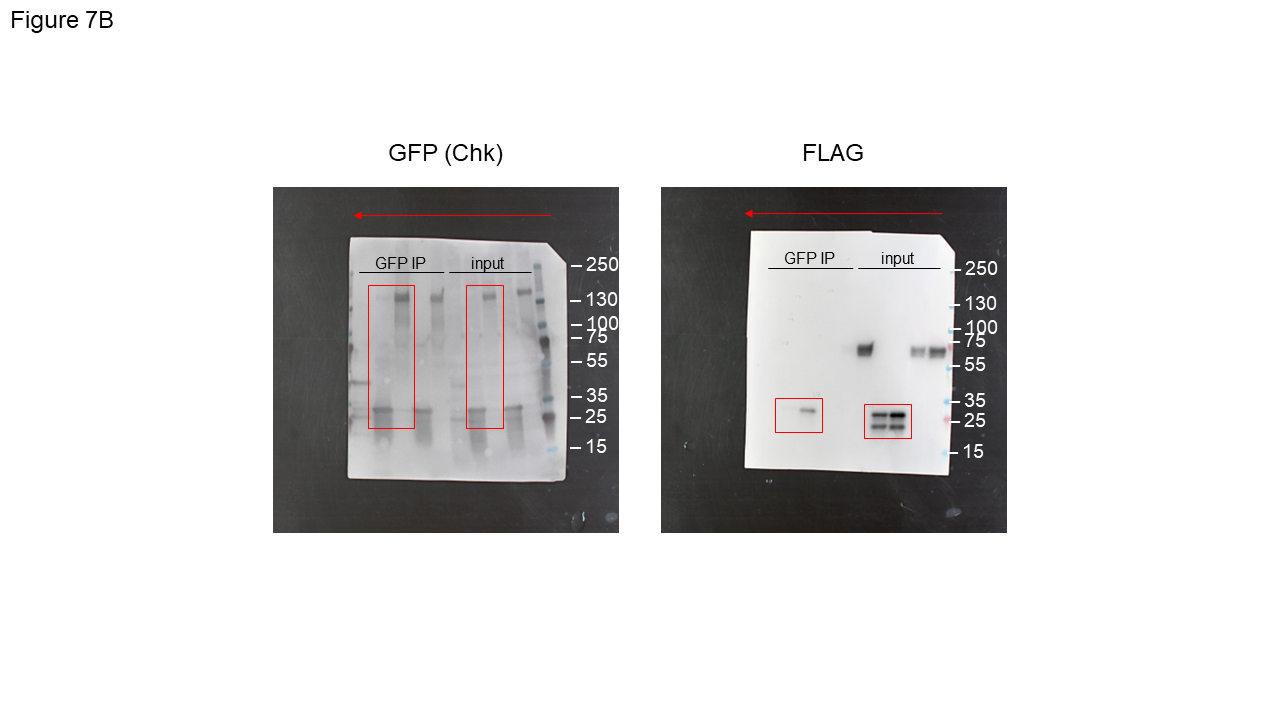

Supplement: Supplementary file 9 — Source data Fig. 7 [file 44319_2024_170_MOESM9_ESM.zip › Figure 7 Source Data/Panel 7B/figure7B_labeling.tif]

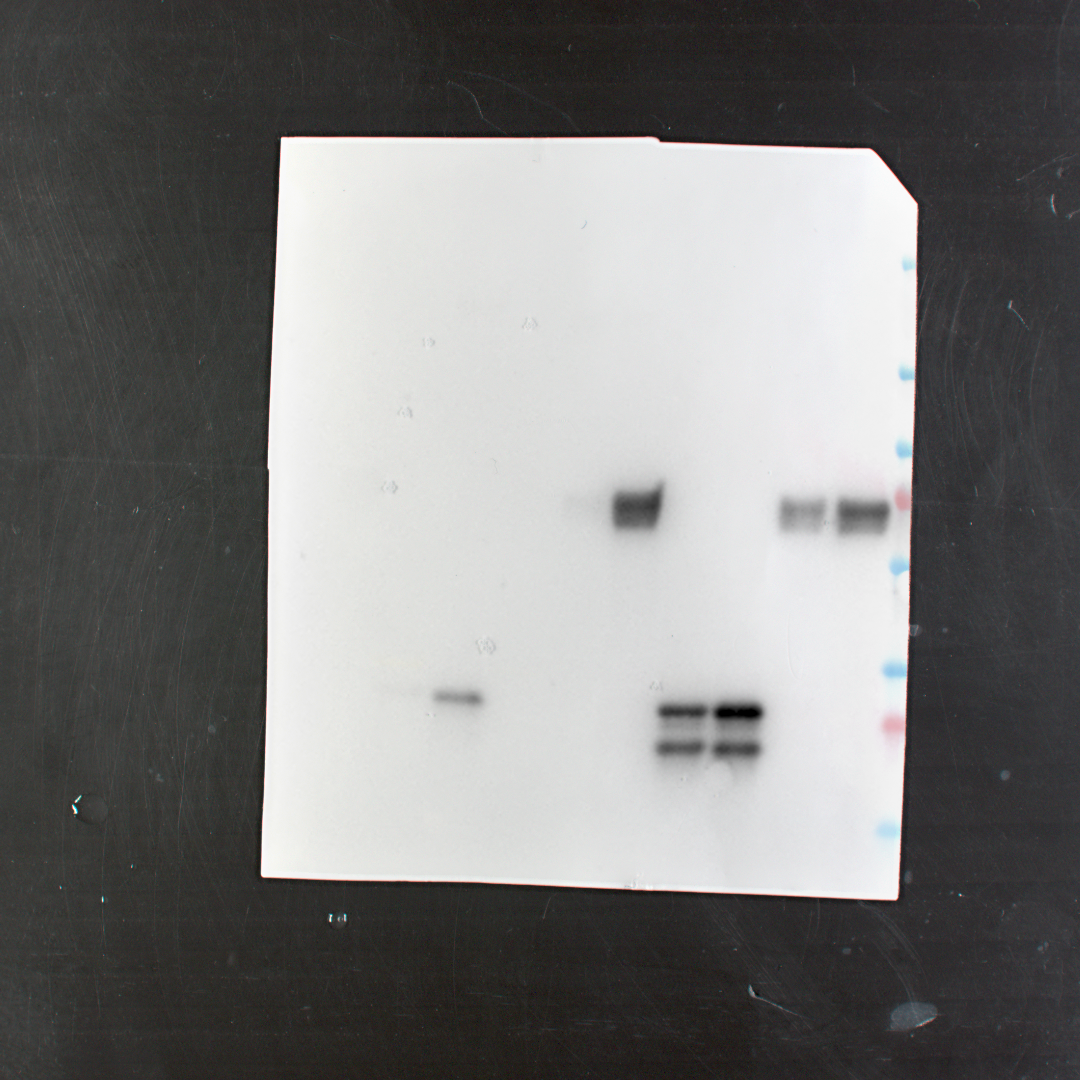

Supplement: Supplementary file 9 — Source data Fig. 7 [file 44319_2024_170_MOESM9_ESM.zip › Figure 7 Source Data/Panel 7B/FLAG_with-ladder.Tif]

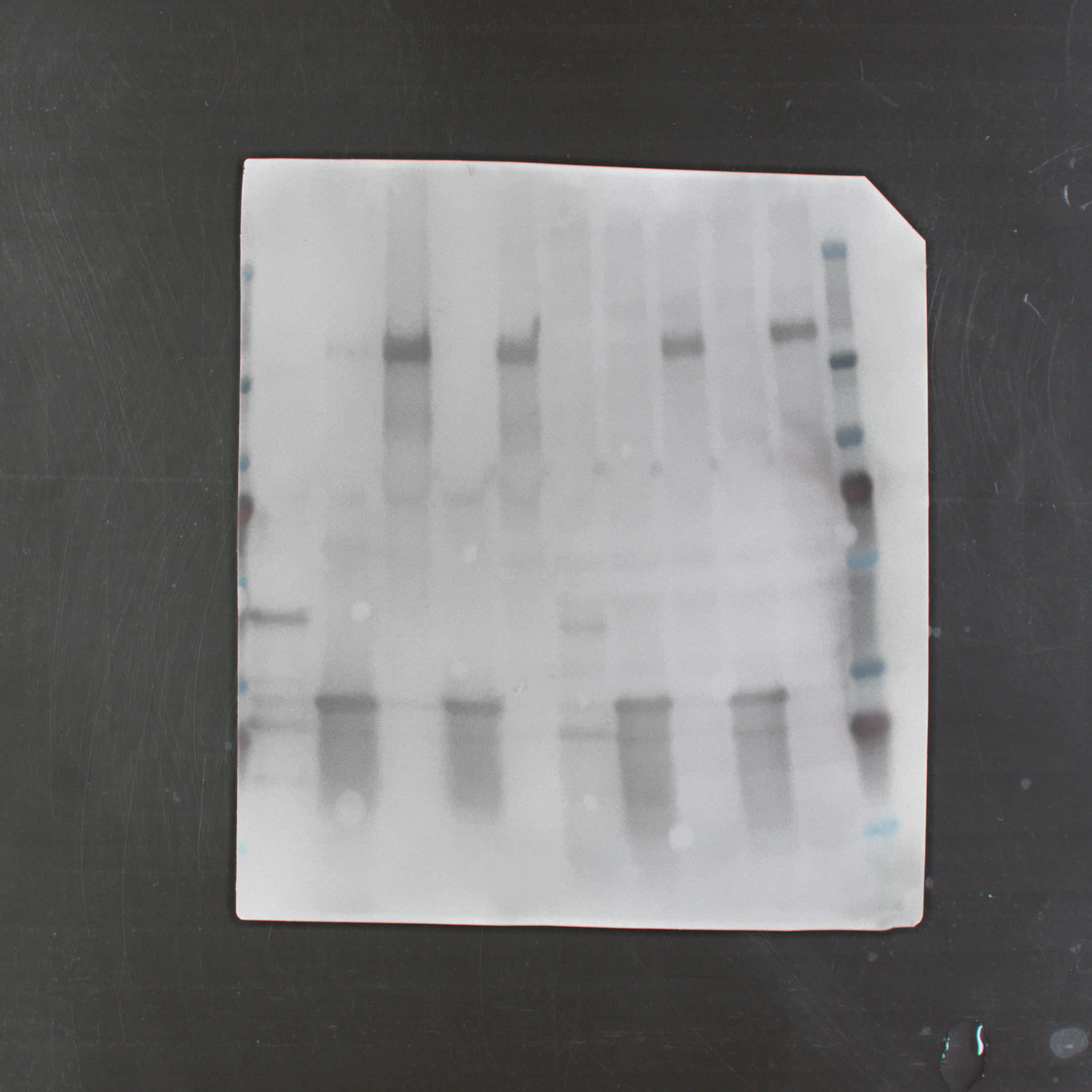

Supplement: Supplementary file 9 — Source data Fig. 7 [file 44319_2024_170_MOESM9_ESM.zip › Figure 7 Source Data/Panel 7B/GFP-Chk-antibody_with-ladder.Tif]
